# *Aspergillus flavus* resident in Kenya: High genetic diversity in an ancient population primarily shaped by clonal reproduction and mutation-driven evolution

**DOI:** 10.1016/j.funeco.2018.05.012

**Published:** 2018-10

**Authors:** Md-Sajedul Islam, Kenneth A. Callicott, Charity Mutegi, Ranajit Bandyopadhyay, Peter J. Cotty

**Affiliations:** a Agricultural Research Service, United States Department of Agriculture, School of Plant Sciences, University of Arizona, Tucson, AZ, USA; b International Institute of Tropical Agriculture, Nairobi, Kenya; c International Institute of Tropical Agriculture, Ibadan, Nigeria

**Keywords:** *Aspergillus flavus*, Aflatoxin biocontrol, Simple sequence repeat (SSR) marker, Genetic diversity, Reproduction, Evolution

## Abstract

*Aspergillus flavus* has long been considered to be an asexual species. Although a sexual stage was recently reported for this species from *in vitro* studies, the amount of recombination ongoing in natural populations and the genetic distance across which meiosis occurs is largely unknown. In the current study, genetic diversity, reproduction and evolution of natural *A. flavus* populations endemic to Kenya were examined. A total of 2744 isolates recovered from 629 maize-field soils across southern Kenya in two consecutive seasons were characterized at 17 SSR loci, revealing high genetic diversity (9-72 alleles/locus and 2140 haplotypes). Clonal reproduction and persistence of clonal lineages predominated, with many identical haplotypes occurring in multiple soil samples and both seasons. Genetic analyses predicted three distinct lineages with linkage disequilibrium and evolutionary relationships among haplotypes within each lineage suggesting mutation-driven evolution followed by clonal reproduction. Low genetic differentiation among adjacent communities reflected frequent short distance dispersal.

## 1 Introduction

*Aspergillus* section *Flavi* includes several species of closely related fungi that frequently contaminate crops with aflatoxins. *Aspergillus flavus* is an important member of this section which infects various food and feed crops, including maize, peanut, cottonseed, and tree nuts, both prior to harvest and during storage. This fungal infection often results in contamination of the crop with aflatoxins, highly toxic and carcinogenic polyketide fungal metabolites (Eaton and Gallagher, 1994; Sweeney and Dobson, 1998). Aflatoxins reduce crop values, and consumption of contaminated crops has a severe effect on the health of humans and domestic animals. Ingestion of aflatoxin-contaminated food may lead to immune system suppression, growth retardation, abnormal fetal development, and in cases of severe exposure, hepatitis, liver necrosis, jaundice, abdominal swellings, cancer and rapid death (Gong et al., 2004; Williams et al., 2004; Azziz-Baumgartner et al., 2005).

Aflatoxin contaminates 25% of world food crops, and 4.5 billion people in the developing countries, especially in Africa and Asia, are chronically exposed to aflatoxins in their daily diets (Williams et al., 2004). Several countries in warm regions, especially in sub-Saharan Africa, have focused on efforts to reduce the magnitude of aflatoxin exposure. The most effective method for preventing aflatoxins is a type of biocontrol that uses atoxigenic genotypes of *A. flavus* to alter fungal communities associated with crops so that aflatoxin producers are less common and the potential for contamination is reduced.

*A. flavus* can be divided into two morphotypes, the L- and S-strains, which differ in physiology, genetics, and the consistency of aflatoxin production (Cotty, 1989). Almost all members of the S-strain morphotype produce high levels of aflatoxins (Cotty, 1989); however, atoxigenic genotypes of the L-strain morphotype are frequently identified from crops and soils (Cotty, 1990, 1994b, 1997). Many of these atoxigenic genotypes are effective as biocontrol agents (Ehrlich, 2014). This biological control method has been successfully used in the U.S. for over two decades to reduce aflatoxin in a variety of crops, such as cotton (Cotty, 1994b), groundnut (Dorner et al., 1998), pistachio and maize (Dorner et al., 1999).

Reproductive mode, dispersal ability and adaptive diversities of the fungal population all may impact efficacy and acceptability of biocontrol fungi. *A. flavus* has long been considered an asexual species that only produces mitospores (conidia) and overwintering asexual fruit bodies (sclerotia) (Amaike and Keller, 2011). Although a sexual stage for *A. flavus* has been reported under laboratory conditions (Geiser et al., 1998; Horn et al., 2009a, 2014, 2016; Moore et al., 2009, 2013; Olarte et al., 2010, 2012), the amount of recombination ongoing in natural populations and the genetic distance across which meiosis occurs in *A. flavus* are subjects of debate (Fisher and Henk, 2012; Grubisha and Cotty, 2015; Runa et al., 2015).

Expression of mating type genes at the *mat* loci (*mat1-1* and *mat1-2*) determines sexual reproduction in filamentous fungi. The distribution of *mat* loci among *A. flavus* genotypes suggests this species is an anamorphic form of a heterothallic ascomycete (Debuchy and Turgeon, 2006; Pal et al., 2007; Ramirez-Prado et al., 2008). Although production of sexual structures from laboratory crosses suggest the possibility of ephemeral, cryptic sexual stages for several *Aspergillus* species (Horn et al., 2009a; Horn et al., 2009b; O'Gorman et al., 2009; Olarte et al., 2012), gene flow among vegetative compatibility groups (VCGs) has not been detected in natural populations of *A. flavus* (Grubisha and Cotty, 2010, 2015), and the significant contribution of sexuality to genetic diversity in agricultural environments continues to be questioned (Grubisha and Cotty, 2010, 2015; Horn et al., 2014; Ortega-Beltran et al., 2016). Recombination in fungal populations may, however, occur in the absence of meiosis through a parasexual cycle characterized by heterokaryon formation, nuclear fusion, and mitotic recombination (Pontecorvo, 1956). Indeed, an active parasexual cycle is reported within but not among VCGs in naturally-occurring *A. flavus* populations (Grubisha and Cotty, 2010, 2015).

Simple sequence repeat (SSR) markers, also known as microsatellites, are frequently used for analyzing genetic diversity and reproductive characteristics because of high levels of polymorphism, simple modes of evolution, relative abundance and extensive genome coverage. SSR markers have proven to be valuable tools for estimating genetic diversity and detecting linkage disequilibrium (LD) associated with the absence of sexual reproduction in fungi and oomycetes, including *Puccinia striiformis* (Cheng and Chen, 2014), *Puccinia triticina* (Kolmer et al., 2013), *Phytophthora ramorum* (Vercauteren et al., 2010) and *Pythium sylvaticum* (Weiland et al., 2015).

In the current study, a subset of 17 SSR markers were selected from a previously described set of 24 SSRs (Grubisha and Cotty, 2009) based on reliable amplification within populations collected across wide geographic ranges (Islam et al., 2014, 2015; Picot et al., 2018). These 17 SSR markers were analyzed across natural populations of the *A. flavus* L-strain morphotype resident in soils associated with maize production in southern Kenya to: (1) quantify the genetic and adaptive diversity of *A. flavus* populations; (2) gain insight into the history and mechanisms of *A. flavus* divergence, including reproductive mode, evolution, and dispersal; and (3) determine frequencies and distribution of common genotypes of *A. flavus* in Kenyan agroecosystems.

Kenya is considered a major ‘hotspot’ for aflatoxin exposure (Azziz-Baumgartner et al., 2005). The human population in Kenya depends upon maize for the majority of consumed calories, and maize produced in Kenya is frequently infected by aflatoxin-producing fungi (Probst et al., 2012). Contamination of maize has repeatedly led to lethal aflatoxicosis in Kenya (Azziz-Baumgartner et al., 2005; Probst et al., 2012). Biocontrol technology based on atoxigenic *A. flavus* has been adapted to aflatoxin management for maize in Kenya (Achia, 2011; Probst et al., 2011; Bandyopadhyay et al., 2016). Information on reproductive mode, dispersal, and adaptive diversity within natural populations of *A. flavus* L-strain morphotypes in Kenya could provide insights useful for assessing the potential impacts, applicability, and acceptability of biocontrol fungi in this region.

## 2 Materials and methods

### 2.1 Sample collection

**Fig. 1:**
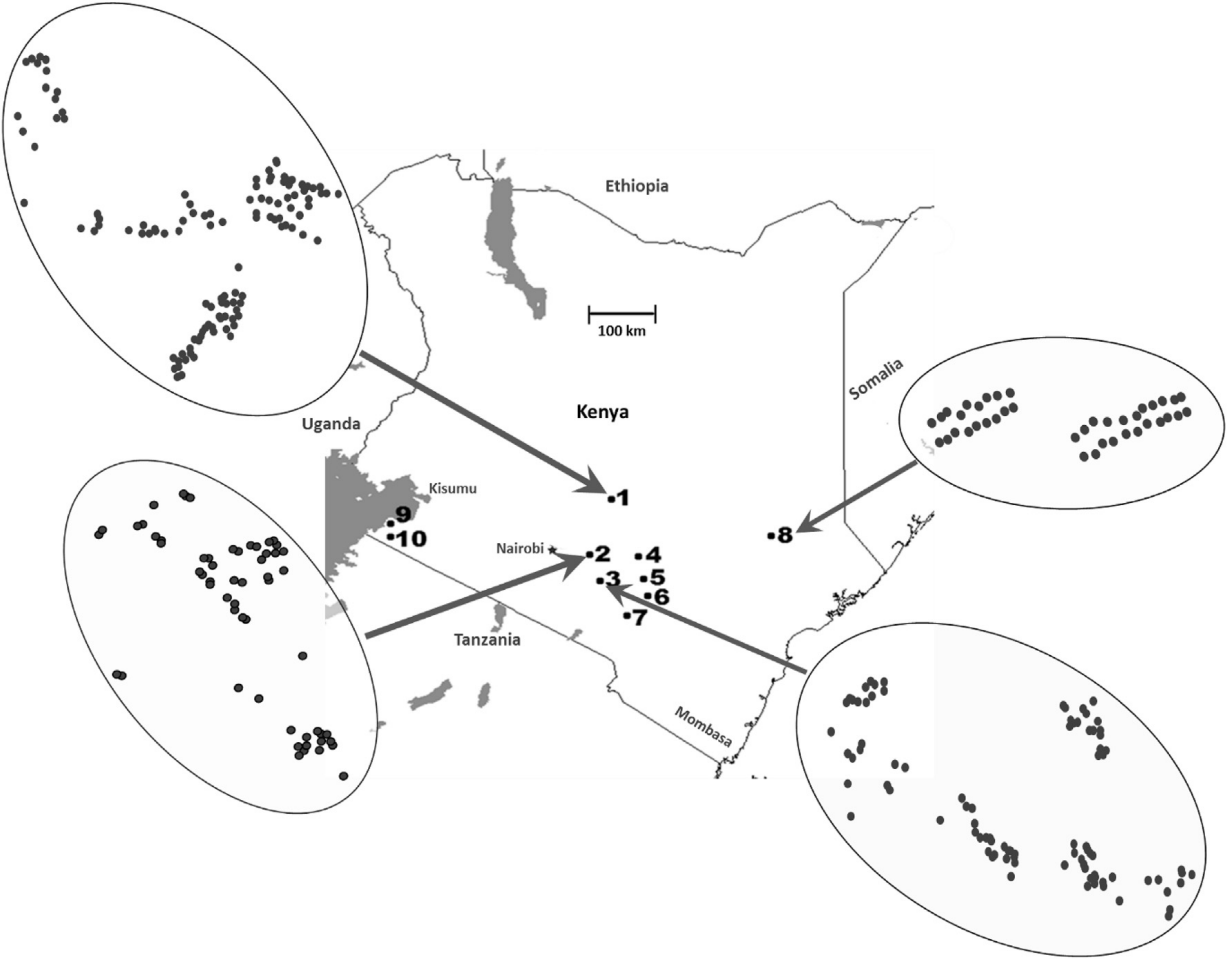
Map of Kenya showing sampling areas and locations. Numbers indicate- locations of the ten agricultural areas in which soils were sampled. Open ovals are enlargements of the four areas in which the most soils were sampled with sample locations indicated by filled circles.

Soil samples from agricultural fields cropped to maize were collected from seven counties (Embu, Machakos, Makueni, Kitui, Tana River, Homabay, and Migori) from southern, southwestern and southeastern Kenya. Sampling was performed during the long rains season (late April to early June) and the short rains season (November to December) during 2012 (Table 1). The two seasons are separated by approximately 6 months. Five counties (Tana River, Makueni, Kitui, Machakos, and Embu) were sampled during the long rains season (May 2012), and six counties (Makueni, Kitui, Machakos, Embu, Homabay and Migori) were sampled during the short rains season (November 2012) (Table 1). Fields were georeferenced prior to sampling. To compare populations resident in regions, ten agricultural areas were delineated across the seven counties (Fig. 1 and Table 1). Soil samples were collected as described previously (Cotty, 1997). Briefly, in each field, 10 scoops (1–3 g) of soil were taken along a 30–40 m transect and combined. Individual soil samples were collected from a total of 629 fields, 390 fields during the long rains season, and 239 during the short season rains (Fig. 1 and Table 1). All samples were shipped to the USDA-ARS-Food and Feed Safety Research Laboratory, Tucson, Arizona, USA under a USDA-APHIS “Permit to Move Live Plant Pests, Noxious Weeds, and Soil.”

**Table 1:** Quantities of *Aspergillus flavus* L-strain morphotype recovered from maize field soils from ten agricultural areas in seven counties in southern, southeastern, and southwestern Kenya during the 2012 long rains and short rains seasons.

Sampling Details			Long Rains Season		Short Rains Season	
Agricultural Area	District/Location	County	Soils^a^ (#)	*A. flavus*^b^ (#)	Soils^a^ (#)	*A. flavus*^b^ (#)
Area-1	Embu East; Mbeere North	Embu	121	343	60	176
Area-2	Kangundo; Kathiani; Matungulu	Machakos	63	275	32	114
Area-3	Machakos; Makueni; Mbooni East	Machakos; Makueni	92	296	70	194
Area-4	Kitui Central; Nzambani	Kitui	40	168	31	154
Area-5	Mutomo	Kitui	–	–	10	86
Area-6	Ikutha	Kitui	14	134	10	90
Area-7	Makindu	Makueni	19	149	10	38
Area-8	Tana River North	Tana River	40	474	–	–
Area-9	Homabay	Homabay	–	–	8	31
Area-10	Rongo	Migori	–	–	8	22
**Total (10 Areas, 16 locations; 7 Counties)**			**390**	**1839**	**239**	**905**

a Number of maize fields sampled. A single composite of soil sample, composed of 30–40 subsamples, were taken from each maize field.

b Number of isolates belonging to the *A. flavus* L-strain morphotype.

### 2.2 Isolation of fungi

Soil samples were subjected to dilution plate technique on the semi-selective modified Rose Bengal agar (M-RB) (Cotty, 1994a). Briefly, the soil was suspended in 0.1% TWEEN-80 solution and spread on M-RB in 9 cm Petri plates. After incubation at 31 °C for 3 d, these colonies were sub-cultured onto 5/2 agar [(5% V-8 juice (Campbell Soup Company, Camden, NJ), 20 g/l bacto-agar, pH 6.0] at 31 °C (Cotty, 1989). A total of 10–15 *Aspergillus* section *Flavi* isolates were cultured from each soil sample. A total of 2744 isolates of *A. flavus* were identified on the basis of colony characteristics and spore morphology. All *A. flavus* belonged to the L-strain morphotype (Table 1). Other *Aspergillus* section *Flavi* isolates belonged to the unnamed S-strain morphology species associated with lethal aflatoxicosis in Kenya (Probst et al., 2012), *Aspergillus tamarii* and *Aspergillus parasiticus*. Isolates were stored as 3-mm plugs of 5 d old cultures on 5/2 agar in sterile distilled water at 4 °C until further use. For long-term storage, select cultures were stored on silica gel.

### 2.3 DNA extraction and SSR genotyping

DNA from each of the 2744 isolates of the *A. flavus* L*-*strain morphotype was extracted with a previously described protocol (Callicott and Cotty, 2015). Briefly, spores were harvested in lysis buffer from agar plates and lysed using heat and mechanical agitation. DNA was precipitated from the lysate, dissolved in water and quantified using a spectrophotometer.

All DNA samples were amplified and genotyped using 17-reliably amplified SSR markers (Table 2). Five multiplex PCR panels were optimized for high-throughput SSR amplifications and genotyping (Table 2). Multiplex PCRs were carried out using 0.08 μmol l^−1^of each primer, 1X AccuStart II PCR SuperMix (Quanta Biosciences, Gaithersburg, MD, USA) and 5 ng of genomic DNA in a final reaction volume of 10 μl. Thermocycler conditions were: 94 °C for 1 min, 26 cycles of 94 °C for 30 s, 57 °C for 90 s, 72 °C for 30 s, and 30 min at 60 °C. PCR amplicons were sized on an ABI 3730 DNA Analyzer at the University of Arizona Core Genomics Laboratory with the LIZ500 standard (Applied Biosystems). Allele sizes were called using GeneMarker version 2.6 (SoftGenetics LLC).

**Table 2:** Diversity of 17 SSR markers across 2744 isolates of the *A. flavus* L-strain morphotype recovered from maize field soils in Kenya during 2012.

PCR^a^ Panel	SSR Locus	Chromosome^b^	Repeat Motif and Scaffold (Grubisha and Cotty, 2009)	Size Range^c^ (bp)	Alleles^d^ (#)	Diversity^e^ (H)
A	AF28	1L	(TTG)_11_/2504	110–192	20	0.889
	AF13	4U	(CTT)_9_/1866	115–204	28	0.898
	AF43	7U	(GAG)_13_/2634	360–426	22	0.863
	AF22	3L	(TTTA)_8_/2911	144–222	18	0.862
	AF31	6U	(TTC)_31_/2634	290–461	44	0.948
B	AF42	6U	(TTC)_16_/2634	139–452	65	0.940
	AF8	3L	(AAG)_16/_2911	144–392	44	0.922
C	AF53	2L	(TCT)_8_/1918	126–213	21	0.660
	AF34	3L	(GTC)_4_ (GTT)_8/_2911	266–434	26	0.860
D	AF16	5L	(TTG)_10_/2541	125–442	55	0.850
	AF54	2L	(ACAT)_8_/1918	128–192	11	0.728
	AF17	2L	(AGA)_4_ (AGG)_10_/1918	330–430	23	0.890
	AF11	1L	(AAG)_12_/2504	106–330	50	0.915
E	AF66	4L	(AT)_12_/1569	184–305	23	0.869
	AF64	2U	(AC)_16_/2856	148–329	72	0.968
	AF63	2U	(AT)_7_/2856	121–144	9	0.684
	AF55	8U	(GT)_10_/1739	159–221	29	0.909

a SSR loci were amplified in multiplex PCR reactions (Panel A through E) which amplified 2 to 5 loci and were read on an ABI 3730 DNA Analyzer with the LIZ500 standard (Applied Biosystems).

b Chromosome, where the SSR locus resides based on the reference of *Aspergillus oryzae* genome (Machida et al., 2005). **‘**L’-lower arm of the chromosome and ‘U’- upper arm of the chromosome.

c Ranges of SSR fragment size based on the variation at SSR repeat numbers across the study isolates.

d Number of alleles detected at the SSR locus.

e Haploid Genetic Diversity (H) which is calculated using the program GenAlEx6.5 (Peakall and Smouse, 2012).

To confirm attribution of variations in amplicon lengths to the repeat number of SSRs regions, representative examples of each allele were sequenced using the previously reported primers known to amplify larger regions around each locus (Grubisha and Cotty, 2009).

### 2.4 Analysis of genetic diversity

GenAlEx version 6.5 (Peakall and Smouse, 2012) was used to calculate both locus and area-wide allelic diversity and genetic diversity including the number of alleles, number of private alleles and haploid genetic diversity (H).

Multilocus SSR haplotypes (genotypes) were identified using HAPLOTYPE-ANALYSIS V 1.04 (Eliades and Eliades, 2009). *A. flavus* produces many conidia on a single conidiophore, and the detected incidence of actively sporulating genotypes may be overrepresented in a sample compared to actual frequencies within the soil community. To avoid this problem, sample correction was conducted where each haplotype was only included once (no repeated haplotypes) for each sample (Table 3). These sample corrected data sets were used as the basis for genetic analyses. Haplotype diversity was estimated for two cropping seasons for the year of 2012 (long rains and short rains) both individually and together. Frequencies of haplotypes detected in two or more samples were compared by analysis of variance (ANOVA) with the general linear model procedure of SAS version 9.2 (SAS Institute, Cary, NC). Mean separations were performed on data from comparisons with statistically significant differences (*P* = 0.05) using Tukey's test (Pagano and Gauvreau, 2000).

**Table 3:** Genetic diversity of *A. flavus* L-strain morphotypes in maize field soils across ten agricultural areas in southern, southeastern and southwestern Kenya during 2012.

Area	District/Location	County	N	Nsc	Na	Nh	Nph	E	Hs (A)
1	Embu East; Mbeere North	Embu	519	479	24.6	453	415	0.935	0.858
2	Kangundo; Kathiani; Matungulu	Machakos	389	341	24.4	326	295	0.958	0.851
3	Machakos; Makueni; Mbooni East	Machakos; Makueni	490	420	24.1	383	342	0.859	0.862
4	Kitui Central; Nzambani	Kitui	322	295	22.9	281	256	0.937	0.861
5	Mutomo	Kitui	86	78	15.5	77	68	0.992	0.834
6	Ikutha	Kitui	224	192	19.2	188	168	0.988	0.829
7	Makindu	Makueni	187	154	19.1	149	138	0.974	0.845
8	Tana River North	Tana River	474	402	21.7	356	338	0.796	0.817
9	Homabay	Homabay	31	27	8.0	27	24	1.000	0.715
10	Rongo	Migori	22	20	8.0	20	18	1.000	0.801
**Overall Kenya**			2744	2408	32.9	2140	2140	0.691	0.862

N, The total number of isolates analyzed.

Nsc, Number of isolates after sample correction (removing repeated haplotypes from the same soil sample-).

Na, Average number of alleles across -17 SSR loci.

Nh, Number of haplotypes.

Nph, Number of private haplotypes.

E, Evenness (as an indicator of how evenly haplotypes are divided over the areas) calculated using the poppr package in R (Kamvar et al., 2014).

Hs (A), The average haploid genetic diversity calculated using the program GenAlEx6.5 (Peakall and Smouse, 2012).

Clonal groups (closely related haplotypes evolved through mitotic reproduction and mutation) were identified based on the principle used for determining clonal groups of asexually reproducing bacterial populations using the program eBURST v3 (Feil et al., 2004). A user-defined group definition was set to include groups with common alleles for at least 14 of the 17 SSR loci.

### 2.5 Analysis of genetic structure, reproductive characteristics and evolution

A multivariate clustering analysis was conducted based on the discriminant analysis of principal components (DAPC) with ADEGENET package in R (Jombart et al., 2010). In DAPC, numbers of clusters were identified based on the Bayesian Information Criterion (BIC) (Jombart et al., 2010). Genetic differentiation among DAPC clusters was further estimated both by pairwise *F*_ST_ (based on infinite allele model, IAM) (Weir and Cockerham, 1984) and *R*_ST_ (based on stepwise mutation model; SMM) (Rousset, 1996) using GENEPOP web version 3.4 (Raymond and Rousset, 1995). *F*_ST_ and *R*_ST_ values were computed on the same data to provide insights on causes of population differentiation, i.e., drift vs. mutation (Ross et al., 1997; Hardy et al., 2003).

Population expansion and evolution of each cluster revealed from DAPC were assessed based on the analysis of linkage disequilibrium (LD) and minimum spanning (MSN) network. LD among the members within each cluster was examined by three different approaches. First, genotypes obtained from the combination of alleles from each pair of SSR loci across the members within each cluster were visualized in three-dimensional surface plots to examine the distributions of genotypes in a surface of total genotypes. Second, significance of associations of alleles between all pairs of SSR loci (pairwise) and distributions of detected genotypes (random vs. non-random) within each cluster were examined separately using two different methods: (1) Fisher exact test implemented in GENEPOP web version 4.0.10 (Raymond and Rousset, 1995) with default parameters (burn-in period (1,000), 100 batches, and 1000 iterations per batch); and (2) Log-likelihood ratio of G-statistic with FSTAT version 2.9.3 (Goudet, 1995) with sequential Bonferroni corrections for multiple tests (Rice, 1989). Finally, to test the null hypothesis of unlinked loci expected in sexually recombining populations, the index of association (*I*_*A*_) (Brown et al., 1980) and the alternative index, $\bar{r}$_*d*_ (less biased to the number of loci) (Agapow and Burt, 2001) were calculated with the poppr package in R (Kamvar et al., 2014) to assess the multilocus genotypic LD within each cluster; with disequilibrium indicated when *I*_*A*_ and $\bar{r}$_*d*_ differ significantly from 0. The evolutionary relationships among the haplotypes within each of the DAPC clusters were illustrated by constructing a minimum spanning network (MSN) using the program PHYLOViZ (Francisco et al., 2012).

### 2.6 Analysis of genotypic distribution and genetic differentiation

Area-wide haplotypic diversity, including the number of haplotypes, private haplotypes and frequencies of haplotypes displayed by two or more isolates, was measured using the program HAPLOTYPE-ANALYSIS v. 1.04. Evenness (an indicator of how evenly haplotypes are divided over the areas) for each agricultural area was calculated using the poppr package in R (Kamvar et al., 2014).

Genetic differentiations within each agricultural area between two growing seasons, and among areas (overall collections from both seasons) were evaluated by the analysis of molecular variance (AMOVA, ARLEQUIN version 3.0, (Excoffier et al., 2005), using a random effect model with 1000 permutations. Pairwise *F*_ST_ (Weir and Cockerham, 1984) and *R*_ST_ (Rousset, 1996) were calculated using GENEPOP web version 3.4 (Raymond and Rousset, 1995) to examine the level of genetic differentiation within and between areas.

## 3 Results

### 3.1 Genetic diversity

SSRs amplifications across the study isolates were free of PCR artifacts and generated single peaks in the expected size range. There were no missing data and no null alleles. Some SSR loci exhibited very large variation in fragment size. For example, AF42 alleles ranged from 139 to 442 bp, and AF16 ranged from 125 to 442 bp (Table 2; Supplementary File 1). Sequencing of smallest and largest alleles from several of these loci demonstrated that the large variation in fragment size can be explained by variation in repeat copy number alone (Supplementary File 2).

**Fig. 2:**
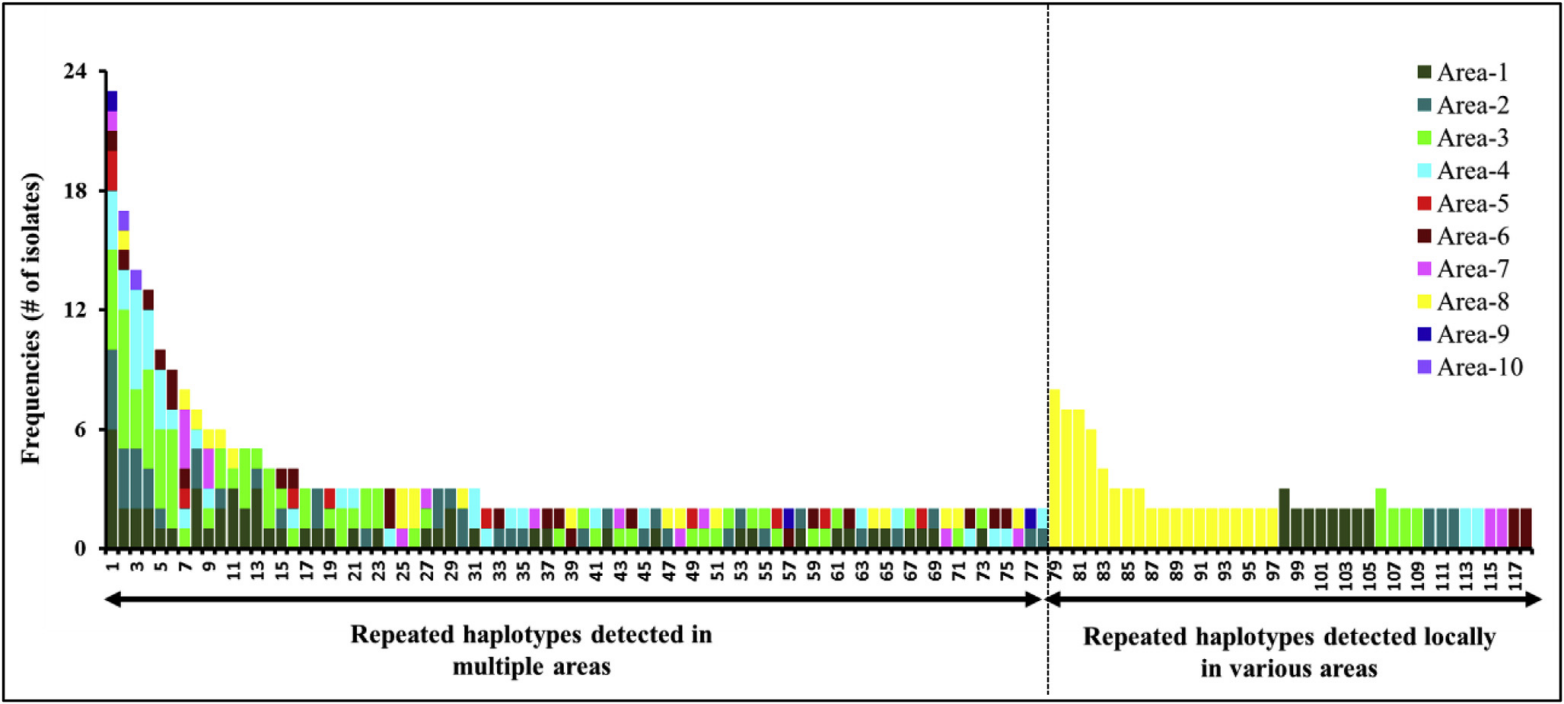
Frequencies of repeated haplotypes (detected two or more times) across 2408 sample corrected isolates of the *A. flavus* L-strain morphotype collected from agricultural soils cropped to maize in ten agricultural areas in Kenya.

Genetic diversity was high with 9–72 alleles, and 0.660 to 0.968 haploid diversity per locus (Table 2). A total of 2140 unique haplotypes were detected among 2744 isolates (Table 3) with 118 haplotypes detected in multiple samples (Fig. 2). The collected isolates were culled to only allow a single isolate with a specific SSR haplotype from each sample. After this sample correction, 2408 isolates remained for population genetic analysis.

In area-wide comparison, observed allelic diversity varied among different agricultural areas, with lower diversity detected in areas with the smallest numbers of samples (Area-5, 9 and 10) (Table 3). However, average haploid genetic diversity among areas was minimal, ranging from 0.715 to 0.862 (Table 3).

Among 2140 haplotypes detected, twenty-seven haplotypes were found in both seasons (Table 4), but detection within an area during one season did not necessarily predict detection in the same area in the second season. The eBURST analysis identified 246 closely related haplotypes (clonal groups) that differed at no more than 3 of 17 loci (> 80% genetic similarity) (Supplementary File 3). The 246 clonal groups accounted for 1541 isolates, leaving 867 haplotypes ungrouped.

**Table 4:** Distributions of repeated haplotypes (displayed by two or more isolates) among *Aspergillus flavus* L-strain morphotype isolates between long rains and short rains seasons during the year of 2012 across ten agricultural areas in Kenya. Numeric values of each column and row from Area-1 to 10 indicates the number of sample corrected isolates with the same haplotype.

Haplotype ID	N	Area-1		Area-2		Area-3		Area-4		Area-5	Area-6		Area-7		Area-8	Area-9	Area-10
		LRS	SRS	LRS	SRS	LRS	SRS	LRS	SRS	SRS	LRS	SRS	LRS	SRS	LRS	SRS	SRS
H-1462	24	3	3	3	1	1	4	–	3	2	–	1	1	–	–	1	–
H-1354	17	–	2	2	1	1	6	–	2	–	–	1	–	–	1	–	1
H-199	15	–	2	–	3	1	2	1	4	–	–	–	–	–	–	–	1
H-1017	13	–	2	1	1	–	5	–	3	–	–	1	–	–	–	–	–
H-212	11	–	1	–	1	–	4	1	2	–	–	1	–	–	–	–	–
H-591	8	–	–	–	–	1	–	1	–	1	1	–	3	–	1	–	–
H-318	6	1	–	–	–	1	–	–	1	–	–	–	2	–	1	–	–
H-557	5	–	3	–	–	–	1	–	–	–	–	–	–	–	1	–	–
H-1019	4	1	–	–	–	–	2	–	–	–	–	–	–	–	–	–	–
H-1158	4	1	–	1	–	1	–	–	–	–	–	1	–	–	–	–	–
H-1302	4	–	–	–	–	–	1	–	1	1	1	–	–	–	–	–	–
H-576	3	–	–	1	–	1	1	–	–	–	–	–	–	–	–	–	–
H-1030	3	1	1	1	–	–	–	–	–	–	–	–	–	–	–	–	–
H-1756	3	1	–	–	–	–	–	1	1	–	–	–	–	–	–	–	–
H-154	3	1	1	–	–	–	–	–	–	–	–	–	–	–	–	–	–
H-155	2	–	–	–	–	–	–	1	–	1	–	–	–	–	–	–	–
H-418	2	–	–	1	–	–	1	–	–	–	–	–	–	–	–	–	–
H-481	2	–	–	–	–	1	–	–	1	–	–	–	–	–	–	–	–
H-532	2	–	–	–	–	–	1	–	–	–	1	–	–	–	–	–	–
H-662	2	–	–	–	–	–	–	1	1	–	–	–	–	–	–	–	–
H-737	2	–	–	–	1	–	–	–	–	–	–	–	–	–	1	–	–
H-1051	2	–	–	–	–	1	–	–	–	1	–	–	–	–	–	–	–
H-1328	2	–	–	–	–	1	–	–	–	1	–	–	–	–	–	–	–
H-1365	2	–	–	1	–	–	–	–	1	–	–	–	–	–	–	–	–
H-1408	2	–	1	–	–	–	–	–	–	–	–	–	–	–	1	–	–
H-1435	2	–	–	–	–	–	–	–	–	–	1	1	–	–	–	–	–
H-1910	2	–	–	–	–	–	–	–	1	–	1	–	–	–	–	–	–

LRS, Long Rains Season.

SRS, Short Rains Season.

H, Haplotypes based on 17-multilocus SSR makers. Only repeated haplotypes detected between two seasons are included.

N, Number repeated haplotypes detected.

### 3.2 Genetic structure, reproduction and evolution

**Fig. 3:**
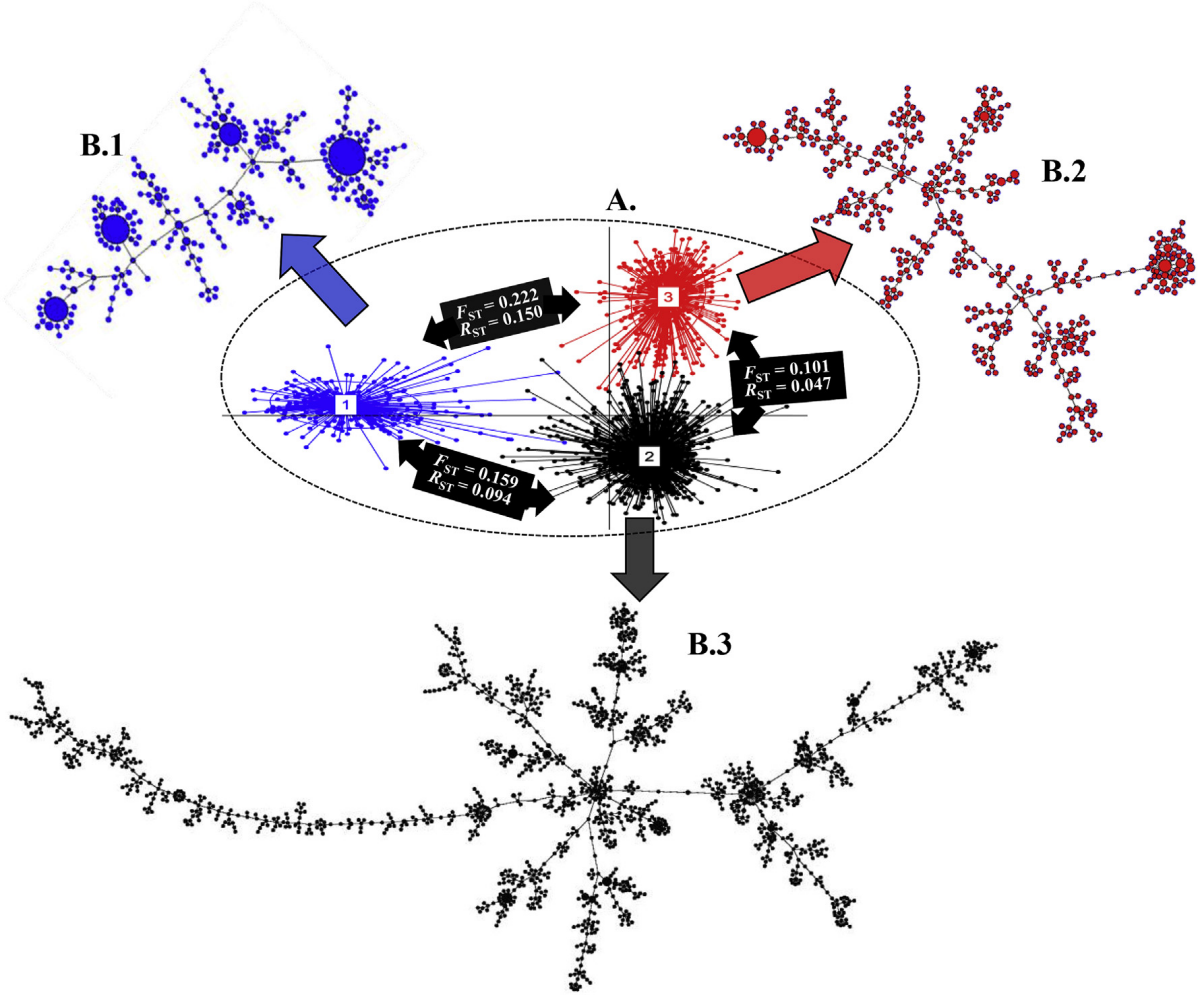
**A.** Scatter-plot of three genetic lineages (based on discriminant analysis of principal component; DAPC) across *A. flavus* L-strain morphotype isolates recovered from ten agricultural areas in Kenya. **B.1, B.2**, and **B.3** are minimum spanning networks for DAPC lineage-1 (349 isolates), lineage-2 (1553 isolates), and lineage-3 (506 isolates).

The discriminant analysis of principal components (DAPC) predicted three genetic clusters (lineages) based on Bayesian information criteria (BIC) (Fig. 3 and Supplementary File 4), where the minimum number of lineages can be predicted at the point after which the BIC increases or decreases by a negligible amount (Jombart et al., 2010). DAPC analyses were repeated ten times, and three lineages were consistently predicted based on BIC (Fig. 3). *F*_ST_ and *R*_ST_ were used to assess the level of genetic differentiation among DAPC lineages and to assess the rates of drift and mutation. When the contribution of stepwise mutation to differentiation is negligible, *R*_ST_ and *F*_ST_ will not significantly differ, while *R*_ST_ will be significantly larger than *F*_ST_ when the rate of stepwise mutation is high (Balloux and Lugon-Moulin, 2002.; Hardy et al., 2003). In all pairwise comparisons between DAPC clusters, *F*_ST_ was larger than *R*_ST_.

**Fig. 4:**
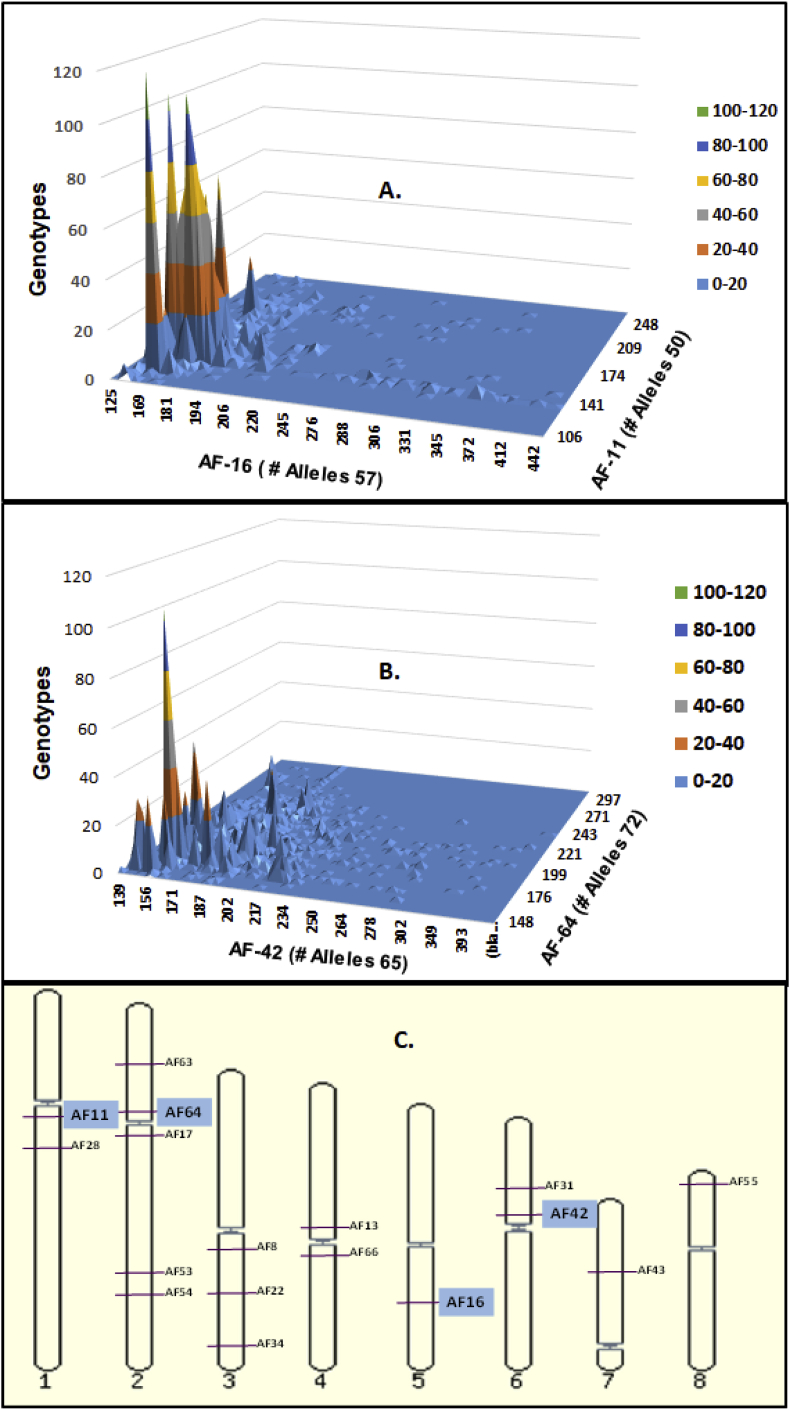
The distributions of genotypes based on allele combinations from two pairs of highly variable SSR loci on the surface of all possible genotypes. **A.** Genotypes based on AF-16 and AF-11; **B.** Genotypes based on AF-42 and AF-64; **C.** Locations of AF-11, AF-64, AF-16 and AF-42 on *A. flavus* chromosomes.

**Fig. 5:**
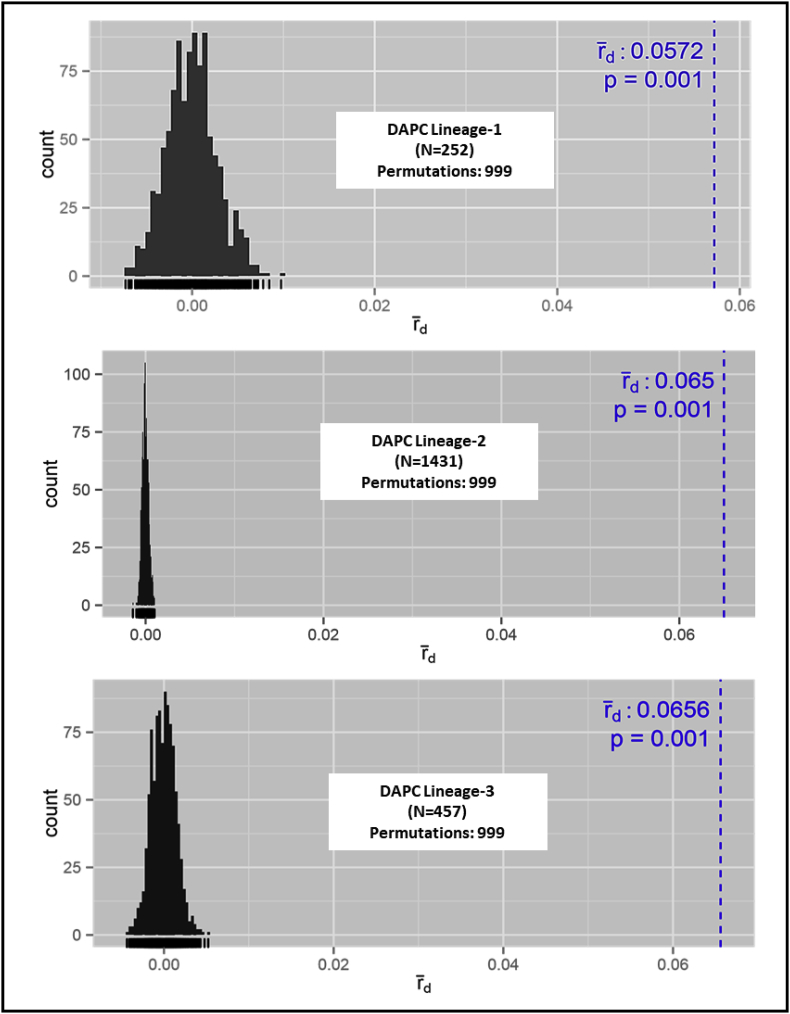
Standardized index of association, $\bar{r}$_*d*_ as the measure of multilocus genotypic linkage disequilibrium (LD) for unique haplotypes (clone corrected data) in each of the three DAPC lineages. The observed $\bar{r}$_*d*_ for each of the DAPC lineages falls outside of the distribution expected under free recombination (dotted blue line). *P* < 0.01 indicates significant LD (at 99.9% level). N, number of unique haplotypes.

Random assortment of alleles among haplotypes within each lineage was tested with three different approaches. First, three-dimensional surface plots for each pair of SSR loci across the members at each lineage revealed clustered distribution of alleles within a narrow range (Fig. 4). Second, allelic associations were tested independently within each DAPC lineage at each pair of SSR loci with data sets composed of all unique haplotypes (i.e., each lineage was clone corrected), and each lineage was found to be in linkage disequilibrium (LD) after Bonferroni correction (in all cases *P* < 0.0004). Third, during the assessment of genotypic equilibrium, both *I*_A_ (data not shown) and ${\bar{r}}_{d}$ differed significantly from 0 (*P* < 0.01) for each lineage (Fig. 5). To minimize the effects of clonal reproduction that might hide signals of infrequent sexual recombination, the ${\bar{r}}_{d}$ analysis was repeated for the dataset as a whole and within each DAPC lineage using single haplotypes randomly selected from each eBURST group (group haplotypes) or using those group haplotypes plus the haplotypes that were not grouped (singletons) during eBURST analysis. In all cases (Supplementary Files 6-9), ${\bar{r}}_{d}$ differed significantly from 0 (*P* < 0.01). Results from these three approaches provide no support for meiotic recombination within any of the lineages. Similarly, the haplotype networks (MSN) bear signatures of clonal reproduction (based on many repeated haplotypes) and mutation-driven evolution (based on relationships among haplotypes) (Fig. 3).

### 3.3 Genotypic distribution and spatial genetic differentiation

In the ten agricultural areas, 70%–82% of isolates had private haplotypes, detected in only one area (Table 3). Even within that narrow range, there was a weak relationship (R^2^ = 0.29) between sample size (number of isolates)and percent isolates with a local distribution (Supplementary File 5). High frequencies of private haplotypes resulted in evenness ranging from 0.798 to 1.000 with 1.000 indicating all haplotypes within an area are equally frequent.

Of 118 haplotypes detected in multiple soils, 78 were detected in multiple areas, and 27 were found in both seasons (Fig. 2 and Table 4). Three haplotypes (H-1462, H-1354 and H-591), were detected in eight, seven and six, respectively, out of the ten agricultural areas (Table 4), and were significantly more widespread than other haplotypes across the study areas (ANOVA, data not shown); however, frequencies did not differ significantly among the other 75 haplotypes that were not locally restricted (Fig. 2). Forty haplotypes were detected in multiple soils but only in a single area (Fig. 2). Sixteen of these were detected in Area-8 which was the area with the greatest number of private haplotypes detected in multiple soils (Fig. 2).

The majority of clonal groups (n = 171 out of 246) detected by eBURST were found in multiple areas, suggesting the wide distribution of these lineages across the study area (Supplementary File 3). Clonal lineages were represented by between 2 and 64 sample corrected isolates or from 2 to 40 unique haplotypes.

AMOVA, *F*_ST,_ and *R*_ST_ consistently revealed very low genetic differentiation between the two seasons for any study area (Table 5). Between season variations ranged from 0.64% to 1.39% (AMOVA), and *F*_ST_ and *R*_ST_ ranged from 0.006 to 0.014 and 0.005 to 0.062, respectively (Table 5). Low genetic variation between seasons allowed for the data from the two seasons to be combined prior to further population genetic analyses.

**Table 5:** Analysis of molecular variance (AMOVA) and *F*_ST_ between *A. flavus* L-strain morphotype isolates from the long rains and short rains seasons during 2012.

Areas	Source of Variation	d.f.	Sum of Squares	Variance Components	Percentage (%) of Variation	*F*_ST_	*R*_ST_
Area-1	LRS Vs SRS	1	24.315	0.0779	1.06	0.011	0.015
Area-2	LRS Vs SRS	1	22.104	0.1019	1.39	0.014	0.006
Area-3	LRS Vs SRS	1	17.954	0.0522	0.71	0.007	0.008
Area-4	LRS Vs SRS	1	15.908	0.0583	0.79	0.008	0.005
Area-6	LRS Vs SRS	1	14.013	0.0758	1.06	0.011	0.013
Area-7	LRS Vs SRS	1	9.567	0.0465	0.64	0.006	0.062

LRS, Long Rains Season.

SRS, Short Rains Season.

Note: Although ten areas were included in the overall study, fields from only six areas were sampled during both seasons.

**Fig. 6:**
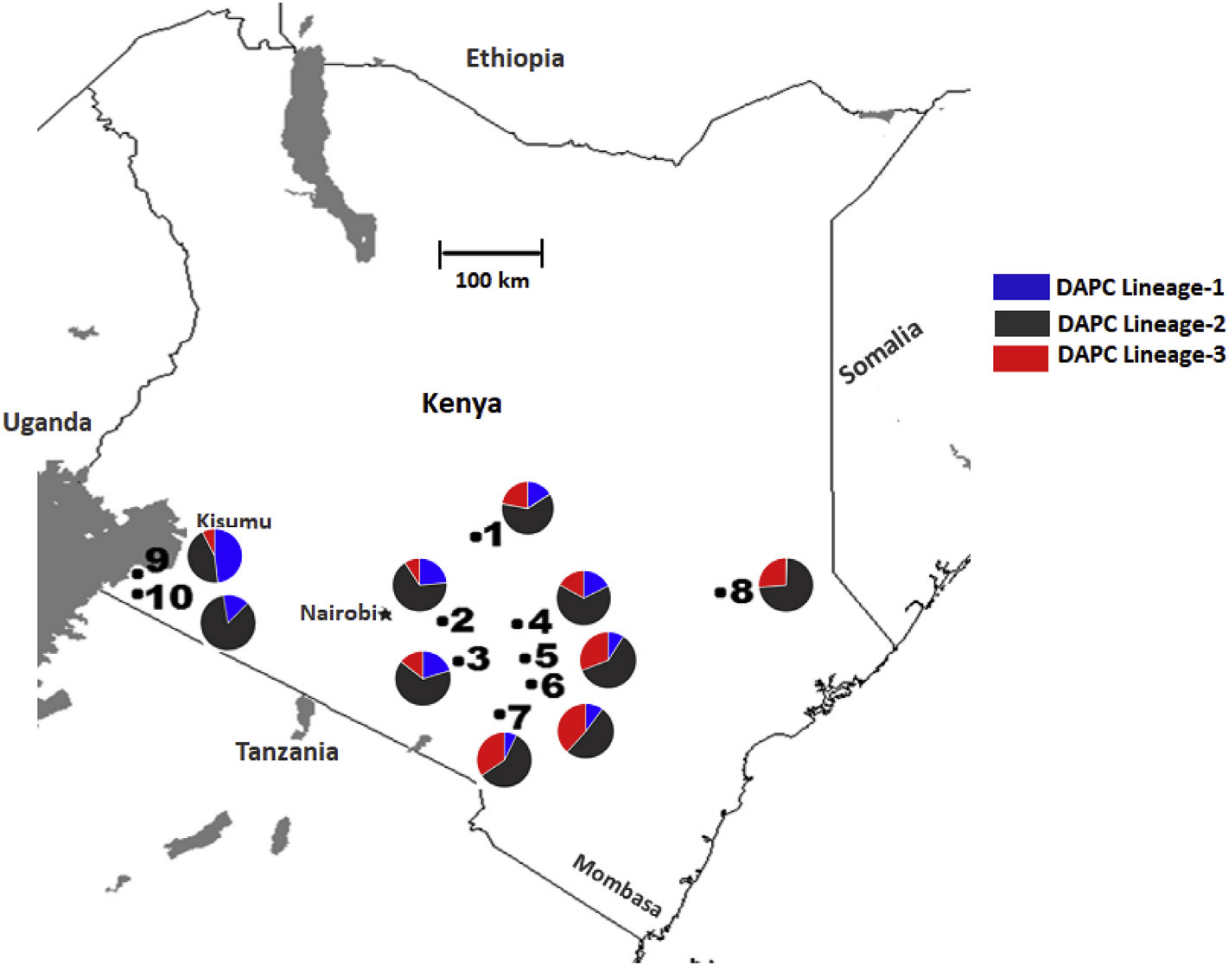
Distributions of three genetic lineages (based on discriminant analysis of principal component; DAPC) of *A. flavus* L-strain morphotype isolates across ten agricultural areas in southern Kenya.

Members of three genetic lineages revealed by DPAC were distributed across Area-1 through Area-7. However, no member from lineage-1 was detected in Area-8, and lineage-3 was less frequent or absent in Area-9 and 10 (Fig. 6). AMOVA detected low genetic variation among areas (1.85%; *F*_ST_ 0.014 and *R*_ST_ 0.019; Table 6) with lower genetic differentiation (based on pairwise *F*_ST_ and *R*_ST_ estimates) among areas in close proximity to each other (Area-1 to 7; Table 7).

**Table 6:** Analysis of molecular variance (AMOVA) among *Aspergillus flavus* L-strain morphotype isolates recovered from maize soil from ten agricultural areas in Kenya during 2012.

Source of variation	d.f.	Sum of squares	Variance components	Percentage of variation	*F*_ST_	*R*_ST_
Among Areas	9	344.982	0.13596 Va	1.85	0.014	0.019
Within Areas	2398	17300.456	7.21454 Vb	98.15

**Table 7:** Pairwise *F*_ST_ (below the diagonal) and *R*_ST_ (above the diagonal) between *Aspergillus flavus* L-strain morphotype isolates recovered from maize field soil from ten agricultural areas in Kenya during 2012.

Agricultural Areas	Area-1	Area-2	Area-3	Area-4	Area-5	Area-6	Area-7	Area-8	Area-9	Area-10
Area-1		0.003	0.004	0.002	0.022	0.009	0.017	0.085	0.062	0.150
Area-2	0.005		0.002	0.003	0.016	0.015	0.008	0.062	0.078	0.171
Area-3	0.002	0.003		0.006	0.027	0.014	0.019	0.088	0.070	0.171
Area-4	0.002	0.004	0.002		0.030	0.014	0.018	0.083	0.066	0.153
Area-5	0.008	0.018	0.009	0.012		0.022	0.001	0.034	0.150	0.239
Area-6	0.011	0.024	0.015	0.014	0.006		0.013	0.080	0.108	0.211
Area-7	0.005	0.014	0.007	0.008	0.002	0.005		0.031	0.135	0.229
Area-8	0.035	0.045	0.035	0.038	0.027	0.036	0.023		0.218	0.308
Area-9	0.045	0.039	0.045	0.044	0.073	0.071	0.067	0.115		0.018
Area-10	0.046	0.043	0.043	0.041	0.072	0.079	0.070	0.102	0.051	

## 4 Discussion

L- and S-strain morphotypes of *A. flavus* differ in several characteristics, including aflatoxin-producing potential and competitive ability during crop colonization (Cotty, 1989; Mehl and Cotty, 2010). Interest in the genetics of the L-morphotype has increased because of the utility of several atoxigenic L-morphotype VCGs as active ingredients in aflatoxin biocontrol products (Mehl et al., 2012; Bandyopadhyay and Cotty, 2013; Atehnkeng et al., 2014; Bandyopadhyay et al., 2016). In the current study, *A. flavus* L-morphotypes were detected in all soils cropped to maize in southern, southwestern and southeastern Kenya (Table 1). The L-morphotype is similarly prevalent in soils and maize kernels across Africa (Tedihou et al., 2012). Although *A. flavus* readily disperses through the air (Bock et al., 2004) it survives in soil as conidia, sclerotia and colonized crop material (Diener et al., 1987; Horn, 2003), with soil reservoirs serving to initiate new cycles of population increase (Jaime-Garcia and Cotty, 2004; Cotty et al., 2008).

### 4.1 Genetic diversity

*A. flavus* exists in communities of thousands of phenotypically diverse VCGs (Bayman and Cotty, 1991; Cotty et al., 1994). Multiple VCGs may occur within a single seed or pinch of soil (Bayman and Cotty, 1991; Mehl and Cotty, 2013), and there is great variability among VCGs in adaptive traits such as aflatoxin-producing ability, virulence, competitiveness, sensitivity to antibiotics, tolerance of soil conditions and life strategy (Bayman and Cotty, 1993; Mehl and Cotty, 2010; Probst et al., 2011). VCG diversity of *A. flavus* populations resident in soil was reported high in all previously examined warm agro-ecologies (Bayman and Cotty, 1991; Horn, 2003), including Kenya (Probst et al., 2011). *A. flavus* VCGs can evolve independently for long periods (Ehrlich et al., 2007; Grubisha and Cotty, 2010), and each VCG can be considered an individual genetic lineage delineated by multiple heterokaryon incompatibility loci (Leslie, 1993). VCG diversity in fungal communities is a primary indicator of genetic diversity (Bayman and Cotty, 1991; Leslie, 1993; Ortega-Beltran and Cotty, 2018). Many highly diverse VCGs shape natural *A. flavus* populations (Bayman and Cotty, 1991; Atehnkeng et al., 2016; Ortega-Beltran and Cotty, 2018). This is consistent with results of the current study where analysis of 17 SSR loci across 2744 isolates detected high genetic diversity. Up to 72 alleles were detected per locus (Table 2), leading to an incredibly diverse community with 2140 haplotypes. VCGs contain individuals with dozens of closely related SSR haplotypes (Grubisha and Cotty, 2010). The high genetic diversity discovered in the current study in *A. flavus* populations in maize soil in Kenya clearly corresponds to high VCG diversity in the fungal community reproducing on maize in Kenya (Probst et al., 2011).

*A. flavus* is ubiquitous in soils of warm regions where it utilizes diverse organic matter including crop debris (Jaime-Garcia and Cotty, 2004). This species may invade grain both during crop development and after maturity (Cotty, 1994b). Moreover, *A. flavus* is not restricted to crops but is a native component of the mycota associated with many plant species and organic debris extending across landscapes and entire agroecosystems. For example, in native desert habitats, *A. flavus* resides in soils and infects and contaminates fruit of leguminous trees (Boyd and Cotty, 2001). Based on this it can be reasonably concluded that *A. flavus* populations in Kenyan soils may have resided in natural habitats for millennia. Many VCGs diverged before the advent of agriculture (Grubisha and Cotty, 2010); in Kenya, the timeline for VCG divergence is likely similar and certainly before the relatively recent introduction of maize to Africa. Allelic diversity is a function of population size, age and stability. High allelic and haplotypic diversity in the study populations consistently suggests *A. flavus* in Kenya has exceptionally large populations that have existed over a very long period with few or no bottlenecks. Along with population size and stability, evolutionary, demographic and historical processes and selection pressures likely contribute to diversity in *A. flavus* populations.

### 4.2 Mode of reproduction and evolution

Results from analyses in the current work suggest the observed genetic diversity arose from the mutation-driven evolution of clonal lineages. Large numbers of alleles (average 32.9 per locus) were detected among 17 SSR loci distributed across the eight *A. flavus* chromosomes (average 32.9); nearly all possible alleles in the ranges for these loci were observed, although the frequencies of many alleles were very low (Supplementary File 1; Fig. 4). Large populations, stable for long periods of time, reach a mutation-drift equilibrium in which many alleles are present at low frequency (Wright, 1966). Therefore, the current results are consistent with a very large and old population.

Further indications of clonal reproduction include detection of haplotypes in multiple samples and across sampling years. Under sexual reproduction even the most likely haplotype (based on frequencies of alleles at each locus) would be expected to be observed 9 × 10^−9^ times in a sample of 2744 isolates, yet 118 of the 2140 observed haplotypes were detected in multiple soil samples, even from as many as eight agricultural areas. The multiple detections of these haplotypes, including detection of 27 haplotypes in both growing seasons, indicates strong dispersal and maintenance of clones through mitotic reproduction. However, these data cannot eliminate the possibility of rare sexual reproduction.

DAPC clusters individuals independent of a priori information (such as geographical location) that may artificially group different genetic lineages introduced in the same area, thus interfering with the detection of migration events (Dutech et al., 2010). Indeed, the number of clusters determined from DAPC may reflect numbers of ancestral lineages. DAPC analysis indicates the diversity detected is best explained by three major genetic clusters (lineages) (Fig. 3). Similarly, haplotype networks (MSN) bear signatures of clonal reproduction (based on repeated haplotypes) and mutation-driven evolution (based on relationships among haplotypes) (Fig. 3). Clonal reproduction and mutation-driven evolution are further supported by significant linkage disequilibrium within clone corrected data (Fig. 5; Supplementary Files 6-9) and the network among haplotypes (MSN in Fig. 3) in each lineage.

Pairwise test of the observed divergence between lineages also supports evolution during clonal reproduction. *R*_ST_ did not exceed *F*_ST_ in any pairwise comparison. This relationship between *R*_ST_ and *F*_ST_ indicates the mutation rate is small relative to the reciprocal of lineage divergence time (Hardy et al., 2003). Each clonal lineage must be very large (to account for the high diversity of alleles at SSR loci) and old (to allow sufficient time for a low mutation rate to generate the observed diversity) (Wright, 1966).

A sexual stage in the *A. flavus* life cycle has been suggested based on sexual structures resulting from crosses and distributions of *mat* loci (Geiser et al., 1998; Horn et al., 2009a, 2014; Moore et al., 2009, 2013; Olarte et al., 2010, 2012; Sweany et al., 2011). Formation of ascocarps can be forced in crosses between species or genera that have diverged for millions of years (Kwon-Chung and Sugui, 2009), and low fertility meiotic crosses between *A. flavus* VCGs have been successful (Horn et al., 2009a). Meiosis in *A. flavus* has been evaluated in laboratory and field plot tests through assessment of a single nuclear segment (∼40 kb of the aflatoxin biosynthesis gene cluster), two SSR loci and mitochondrial DNA (Horn et al., 2016). However, the importance of meiosis in the life cycle is a population genetics question that must take into account multiple generations, not just the F1 and multiple years.

The current study analyzed thousands of *A. flavus* individuals, from across a large area, and revealed no random association of loci within *A. flavus* populations. Earlier studies describing natural *A. flavus* populations also revealed lack of sexual reproduction. Uneven distribution of mating type genes and analysis of genetic data demonstrated strong support for clonal reproduction in *A. flavus* populations from maize field soils in Hungary (Varga et al., 2011). Likewise, studies using over 15 unlinked SSR loci distributed across all eight chromosomes found no evidence for recombination between aflatoxin producers and *A. flavus* AF36, the active ingredient of two EPA registered atoxigenic biocontrol products, after over ten years of commercial use (Ehrlich et al., 2007; Grubisha and Cotty, 2015). Instead, clonal reproduction was mainly observed within *A. flavus* VCG YV36 (AF36) based on the analysis of 237 natural isolates across several southwestern states of the U.S. (Grubisha and Cotty, 2015). Ultimately, it takes analyses of actual populations, like the current study, to determine the extent to which meiosis occurs in *A. flavus*. Such studies, by their nature, include not just production of viable F1 progeny but also long-term survival and competitiveness of subsequent progeny and establishment or loss of clonally evolving lineages.

SSR analyses in the present study suggest that natural populations of *A. flavus* in Kenya are largely evolving in the absence of meiosis through a clonal process. However, parasexual recombination within VCGs could have contributed to the diverse *A. flavus* population in Kenya as previously suggested in *A. flavus* based on linkage equilibrium within but not between VCGs (Grubisha and Cotty, 2010, 2015). VCGs are delimited by multiple polyallelic *het* loci (Leslie, 1993). If evolution is primarily clonal, mutations at *het* loci should result in sister VCGs genetically isolated from each other. On the other hand, even small amounts of sexual recombination among the finite number of *het* loci should allow particular VCGs to arise from different evolutionary backgrounds. This is counter-indicated by the minimum spanning network which exhibited a bifurcating network expected under clonal but not sexual evolution, with each DAPC lineage containing several distinct sublineages comprised of many closely related haplotypes (Fig. 3). The observed branch length variation indicates either varying rates of mutation or loss of less successful intermediate haplotypes (Fig. 3).

### 4.3 Distribution, genetic differentiation and adaptation

Several haplotypes were detected in multiple areas (Fig. 2) and both growing seasons (Table 4). This result suggests the persistence of clonal lineages over seasons. High frequencies, wide distributions and persistence (over seasons) of some haplotypes (e.g., H-1462, H-1354 and H-591) and their close relatives (clonal groups) across the study areas indicate these genotypes are well adapted to Kenyan agro-ecosystems and can be well suited as active ingredients for aflatoxin biocontrol products if atoxigenic. The distribution of haplotypes across the study areas likely resulted from contemporary propagule dispersal, but timings and origins of the dispersal cannot be determined with the current data.

Three genetic lineages were distributed across Area-1 through 7, and population genetic analyses revealed very low genetic differentiation among areas in close proximity to each other (Embu, Kitui, Makueni and Machakos counties) (Table 7). This is consistent with the substantial dispersal of fungal spores among connected areas. Both VCGs and SSR haplotypes of *A. flavus* are widely distributed (Horn, 2003). Several *A. flavus* VCGs isolated from agricultural soils occurred across a large section of the United States and Africa (Horn and Dorner, 1998, 1999; Ehrlich et al., 2007; Grubisha and Cotty, 2010, 2015; Ogunbayo et al., 2013). Dispersal of *A. flavus* is consistent with the production of large quantities of airborne conidia (Bock et al., 2004) and association with both insects (Stephenson and Russell, 1974) and human transported crop materials (Garber and Cotty, 2014). The *A. flavus* communities that reside in Area-8, 9 and 10 (Tana River, Homa Bay and Migori counties) differed from that resident in the other seven areas, possibly due to either physical barriers to dispersal, (i.e., stretches of arid land) or different requirements for adaptive success. Requirements for niche residence in these counties may be partially responsible for higher frequencies of locally restricted haplotypes.

## 5 Conclusion

The genetic diversity observed among *A. flavus* L-strain morphotype isolates resident in maize growing soil in Kenya is attributable to both very high levels of allelic diversity at the SSR loci and a very large and stable population. Genetic analyses suggest that Kenyan soils have very large populations of *A. flavus* with three major founder haplotypes from which the other haplotypes have derived via clonal reproduction and mutation. LD and allelic co-occurrence analyses strongly suggest clonal reproduction and preclude meiotic recombination as a major force shaping *A. flavus* population structure. This result cannot prove that sexual recombination never occurs in nature, but it suggests that influences of meiosis are minor during time-scales over which shifts in agriculture occur. Very low genetic variation among agro-ecologically similar and geographically adjacent areas indicates that populations in these areas are evolving together with high rates of migration.

## Supplementary data

Suplementary File-Islam Suplementary File-Islam
