# Supplementary material for: Aspergillus flavus resident in Kenya: High genetic diversity in an ancient population primarily shaped by clonal reproduction and mutation-driven evolution
Source: Fungal Ecol. 2018 Oct;35:20–33. doi: 10.1016/j.funeco.2018.05.012 (PMC6131765; doi:10.1016/j.funeco.2018.05.012)

**Supplementary File 1**: Allele counts for 17-SSR loci across 2,408 isolates of *A. flavus* L-strain morphology recovered from soils cropped to maize in southern, southeastern and southwestern Kenya

| **^a^AF**  **28** | **N** | **^a^AF**  **13** | **N** | **^a^AF**  **43** | **N** | **^a^AF**  **22** | **N** | **^a^AF**  **31** | **N** | **^a^AF**  **42** | **N** | **^a^AF**  **8** | **N** | **^a^AF**  **53** | **N** | **^a^AF34** | **N** | **^a^AF**  **16** | **N** | **^a^AF54** | **N** | **^a^AF**  **17** | **N** | **^a^AF**  **11** | **N** | **^a^AF**  **66** | **N** | **^a^AF64** | **N** | **^a^AF**  **63** | **N** | **^a^AF**  **55** | **N** |
| --- | --- | --- | --- | --- | --- | --- | --- | --- | --- | --- | --- | --- | --- | --- | --- | --- | --- | --- | --- | --- | --- | --- | --- | --- | --- | --- | --- | --- | --- | --- | --- | --- | --- |
| **110** | **115** | **115** | **54** | **360** | **2** | **144** | **119** | **290** | **49** | **139** | **7** | **144** | **24** | **126** | **13** | **266** | **3** | **125** | **1** | **128** | **1** | **330** | **37** | **106** | **7** | **184** | **2** | **148** | **1** | **121** | **6** | **159** | **22** |
| **113** | **343** | **122** | **21** | **365** | **50** | **155** | **15** | **293** | **60** | **143** | **26** | **147** | **14** | **128** | **166** | **285** | **5** | **141** | **1** | **142** | **1** | **334** | **10** | **109** | **7** | **194** | **5** | **153** | **9** | **123** | **1** | **162** | **58** |
| **116** | **30** | **125** | **110** | **367** | **20** | **158** | **4** | **296** | **3** | **146** | **107** | **151** | **150** | **131** | **121** | **290** | **13** | **161** | **105** | **145** | **63** | **336** | **3** | **114** | **3** | **198** | **46** | **156** | **40** | **125** | **52** | **164** | **53** |
| **119** | **524** | **128** | **70** | **370** | **284** | **163** | **151** | **299** | **150** | **150** | **50** | **154** | **76** | **134** | **1507** | **293** | **86** | **165** | **10** | **153** | **2** | **339** | **30** | **117** | **5** | **201** | **60** | **159** | **38** | **127** | **1410** | **167** | **28** |
| **122** | **73** | **132** | **8** | **373** | **152** | **168** | **8** | **302** | **13** | **153** | **18** | **157** | **32** | **137** | **75** | **296** | **93** | **169** | **527** | **157** | **25** | **342** | **3** | **120** | **8** | **232** | **1** | **161** | **77** | **129** | **277** | **170** | **119** |
| **125** | **112** | **135** | **347** | **376** | **67** | **173** | **18** | **305** | **49** | **156** | **225** | **160** | **91** | **140** | **4** | **298** | **56** | **172** | **149** | **161** | **953** | **344** | **17** | **123** | **157** | **248** | **3** | **163** | **59** | **131** | **494** | **172** | **197** |
| **128** | **33** | **138** | **274** | **379** | **613** | **176** | **50** | **309** | **276** | **159** | **359** | **163** | **85** | **144** | **480** | **301** | **819** | **175** | **554** | **165** | **133** | **347** | **25** | **126** | **29** | **251** | **9** | **165** | **104** | **133** | **284** | **174** | **432** |
| **131** | **374** | **141** | **560** | **382** | **240** | **179** | **501** | **312** | **139** | **162** | **310** | **166** | **273** | **147** | **12** | **304** | **118** | **178** | **617** | **169** | **187** | **350** | **262** | **129** | **35** | **253** | **223** | **167** | **43** | **135** | **217** | **176** | **233** |
| **135** | **403** | **145** | **307** | **385** | **638** | **184** | **318** | **315** | **55** | **165** | **63** | **168** | **434** | **151** | **213** | **307** | **331** | **181** | **325** | **172** | **1006** | **353** | **566** | **132** | **91** | **255** | **179** | **169** | **216** | **137** | **4** | **178** | **441** |
| **138** | **289** | **148** | **164** | **387** | **299** | **188** | **666** | **318** | **240** | **168** | **33** | **171** | **457** | **154** | **46** | **310** | **145** | **184** | **157** | **176** | **295** | **356** | **285** | **135** | **46** | **257** | **26** | **171** | **177** | **139** | **1** | **180** | **359** |
| **142** | **174** | **151** | **77** | **390** | **89** | **192** | **314** | **322** | **136** | **171** | **146** | **174** | **127** | **157** | **45** | **314** | **346** | **188** | **8** | **180** | **50** | **359** | **382** | **138** | **487** | **259** | **13** | **174** | **47** | **144** | **2** | **182** | **95** |
| **145** | **79** | **155** | **121** | **393** | **48** | **196** | **208** | **325** | **39** | **174** | **88** | **177** | **108** | **160** | **7** | **317** | **303** | **191** | **56** | **184** | **26** | **362** | **194** | **141** | **450** | **261** | **307** | **176** | **44** |  |  | **184** | **110** |
| **148** | **34** | **158** | **189** | **396** | **32** | **200** | **276** | **328** | **19** | **178** | **84** | **180** | **50** | **163** | **31** | **320** | **131** | **194** | **61** | **192** | **6** | **365** | **136** | **144** | **258** | **263** | **82** | **178** | **78** |  |  | **186** | **21** |
| **152** | **28** | **161** | **237** | **399** | **88** | **204** | **24** | **331** | **236** | **181** | **160** | **183** | **33** | **166** | **6** | **323** | **90** | **197** | **17** |  |  | **368** | **302** | **147** | **133** | **265** | **24** | **180** | **139** |  |  | **189** | **61** |
| **155** | **11** | **164** | **70** | **402** | **45** | **208** | **59** | **334** | **117** | **184** | **148** | **186** | **62** | **169** | **4** | **326** | **33** | **200** | **2** |  |  | **371** | **221** | **150** | **162** | **267** | **250** | **183** | **152** |  |  | **191** | **55** |
| **158** | **46** | **167** | **22** | **405** | **44** | **213** | **10** | **337** | **234** | **187** | **55** | **189** | **64** | **172** | **2** | **330** | **15** | **203** | **14** |  |  | **374** | **90** | **153** | **133** | **269** | **773** | **185** | **72** |  |  | **193** | **74** |
| **161** | **40** | **170** | **12** | **408** | **9** | **216** | **6** | **340** | **45** | **190** | **42** | **191** | **119** | **175** | **5** | **333** | **83** | **206** | **7** |  |  | **377** | **22** | **156** | **59** | **271** | **303** | **187** | **65** |  |  | **195** | **150** |
| **165** | **35** | **173** | **5** | **411** | **3** | **222** | **1** | **343** | **83** | **193** | **222** | **194** | **98** | **178** | **5** | **336** | **53** | **210** | **8** |  |  | **379** | **8** | **159** | **105** | **273** | **205** | **189** | **81** |  |  | **197** | **126** |
| **174** | **4** | **176** | **32** | **414** | **18** |  |  | **346** | **60** | **196** | **113** | **197** | **44** | **182** | **2** | **339** | **8** | **213** | **9** |  |  | **382** | **130** | **162** | **90** | **275** | **193** | **191** | **112** |  |  | **199** | **22** |
| **192** | **1** | **179** | **24** | **418** | **5** |  |  | **349** | **142** | **199** | **48** | **200** | **17** | **185** | **1** | **342** | **6** | **216** | **11** |  |  | **385** | **18** | **165** | **121** | **277** | **11** | **193** | **91** |  |  | **201** | **14** |
|  |  | **182** | **14** | **421** | **1** |  |  | **352** | **116** | **202** | **80** | **203** | **57** | **213** | **3** | **345** | **1** | **220** | **8** |  |  | **388** | **5** | **168** | **44** | **279** | **27** | **195** | **105** |  |  | **203** | **14** |
|  |  | **185** | **3** | **426** | **1** |  |  | **355** | **27** | **205** | **31** | **206** | **86** |  |  | **352** | **4** | **223** | **8** |  |  | **390** | **1** | **171** | **55** | **284** | **3** | **197** | **77** |  |  | **205** | **6** |
|  |  | **188** | **7** |  |  |  |  | **358** | **39** | **208** | **20** | **209** | **99** |  |  | **407** | **3** | **229** | **1** |  |  | **430** | **1** | **174** | **14** | **286** | **2** | **199** | **29** |  |  | **207** | **13** |
|  |  | **191** | **6** |  |  |  |  | **361** | **90** | **211** | **32** | **212** | **31** |  |  | **415** | **1** | **239** | **3** |  |  |  |  | **177** | **17** | **305** | **1** | **201** | **26** |  |  | **210** | **10** |
|  |  | **194** | **9** |  |  |  |  | **364** | **38** | **214** | **22** | **215** | **51** |  |  | **422** | **1** | **245** | **1** |  |  |  |  | **180** | **13** |  |  | **203** | **32** |  |  | **212** | **17** |
|  |  | **197** | **2** |  |  |  |  | **367** | **66** | **217** | **39** | **218** | **7** |  |  | **434** | **1** | **255** | **1** |  |  |  |  | **183** | **25** |  |  | **205** | **39** |  |  | **214** | **5** |
|  |  | **200** | **2** |  |  |  |  | **370** | **33** | **220** | **42** | **221** | **16** |  |  |  |  | **264** | **3** |  |  |  |  | **186** | **5** |  |  | **207** | **48** |  |  | **217** | **5** |
|  |  | **204** | **1** |  |  |  |  | **373** | **79** | **223** | **39** | **224** | **8** |  |  |  |  | **273** | **3** |  |  |  |  | **189** | **7** |  |  | **209** | **31** |  |  | **219** | **2** |
|  |  |  |  |  |  |  |  | **376** | **45** | **227** | **15** | **227** | **2** |  |  |  |  | **276** | **1** |  |  |  |  | **192** | **12** |  |  | **211** | **57** |  |  | **221** | **6** |
|  |  |  |  |  |  |  |  | **379** | **26** | **231** | **14** | **230** | **3** |  |  |  |  | **279** | **2** |  |  |  |  | **195** | **3** |  |  | **213** | **59** |  |  |  |  |
|  |  |  |  |  |  |  |  | **382** | **7** | **234** | **9** | **233** | **5** |  |  |  |  | **282** | **7** |  |  |  |  | **200** | **7** |  |  | **215** | **50** |  |  |  |  |
|  |  |  |  |  |  |  |  | **385** | **12** | **237** | **7** | **236** | **4** |  |  |  |  | **285** | **5** |  |  |  |  | **203** | **33** |  |  | **217** | **63** |  |  |  |  |
|  |  |  |  |  |  |  |  | **388** | **5** | **241** | **5** | **239** | **6** |  |  |  |  | **288** | **7** |  |  |  |  | **206** | **9** |  |  | **219** | **60** |  |  |  |  |
|  |  |  |  |  |  |  |  | **391** | **4** | **244** | **4** | **242** | **1** |  |  |  |  | **291** | **2** |  |  |  |  | **209** | **23** |  |  | **221** | **21** |  |  |  |  |
|  |  |  |  |  |  |  |  | **394** | **1** | **247** | **4** | **247** | **2** |  |  |  |  | **294** | **5** |  |  |  |  | **212** | **15** |  |  | **223** | **16** |  |  |  |  |
|  |  |  |  |  |  |  |  | **397** | **2** | **250** | **5** | **256** | **2** |  |  |  |  | **297** | **2** |  |  |  |  | **215** | **14** |  |  | **225** | **33** |  |  |  |  |
|  |  |  |  |  |  |  |  | **399** | **3** | **253** | **3** | **259** | **2** |  |  |  |  | **306** | **4** |  |  |  |  | **218** | **18** |  |  | **227** | **57** |  |  |  |  |
|  |  |  |  |  |  |  |  | **403** | **1** | **256** | **2** | **261** | **2** |  |  |  |  | **309** | **4** |  |  |  |  | **221** | **9** |  |  | **229** | **20** |  |  |  |  |
|  |  |  |  |  |  |  |  | **406** | **1** | **259** | **8** | **282** | **1** |  |  |  |  | **323** | **1** |  |  |  |  | **225** | **8** |  |  | **231** | **17** |  |  |  |  |
|  |  |  |  |  |  |  |  | **409** | **1** | **262** | **2** | **294** | **1** |  |  |  |  | **328** | **2** |  |  |  |  | **228** | **11** |  |  | **233** | **27** |  |  |  |  |
|  |  |  |  |  |  |  |  | **425** | **1** | **264** | **4** | **303** | **1** |  |  |  |  | **331** | **6** |  |  |  |  | **230** | **2** |  |  | **235** | **16** |  |  |  |  |
|  |  |  |  |  |  |  |  | **429** | **2** | **267** | **3** | **353** | **1** |  |  |  |  | **335** | **1** |  |  |  |  | **233** | **4** |  |  | **237** | **23** |  |  |  |  |
|  |  |  |  |  |  |  |  | **450** | **2** | **270** | **2** | **361** | **1** |  |  |  |  | **338** | **1** |  |  |  |  | **236** | **1** |  |  | **239** | **29** |  |  |  |  |
|  |  |  |  |  |  |  |  | **461** | **2** | **273** | **2** | **392** | **1** |  |  |  |  | **342** | **2** |  |  |  |  | **239** | **1** |  |  | **241** | **15** |  |  |  |  |
|  |  |  |  |  |  |  |  |  |  | **276** | **4** |  |  |  |  |  |  | **345** | **2** |  |  |  |  | **248** | **2** |  |  | **243** | **31** |  |  |  |  |
|  |  |  |  |  |  |  |  |  |  | **278** | **4** |  |  |  |  |  |  | **348** | **3** |  |  |  |  | **253** | **1** |  |  | **245** | **10** |  |  |  |  |
|  |  |  |  |  |  |  |  |  |  | **281** | **4** |  |  |  |  |  |  | **354** | **7** |  |  |  |  | **259** | **2** |  |  | **247** | **9** |  |  |  |  |
|  |  |  |  |  |  |  |  |  |  |  |  |  |  |  |  |  |  |  |  |  |  |  |  |  |  |  |  | **Continued to next page** | | | | |  |
| **^a^AF**  **28** | **N** | **^a^AF**  **13** | **N** | **^a^AF**  **43** | **N** | **^a^AF**  **22** | **N** | **^a^AF**  **31** | **N** | **^a^AF**  **42** | **N** | **^a^AF**  **8** | **N** | **^a^AF**  **53** | **N** | **^a^AF34** | **N** | **^a^AF**  **16** | **N** | **^a^AF54** | **N** | **^a^AF**  **17** | **N** | **^a^AF**  **11** | **N** | **^a^AF**  **66** | **N** | **^a^AF64** | **N** | **^a^AF**  **63** | **N** | **^a^AF**  **55** | **N** |
|  |  |  |  |  |  |  |  |  |  |  |  |  |  |  |  |  |  |  |  |  |  |  |  |  |  |  |  |  |  |  |  |  |  |
|  |  |  |  |  |  |  |  |  |  | **290** | **11** |  |  |  |  |  |  | **363** | **4** |  |  |  |  | **289** | **2** |  |  | **250** | **8** |  |  |  |  |
|  |  |  |  |  |  |  |  |  |  | **293** | **4** |  |  |  |  |  |  | **372** | **2** |  |  |  |  | **303** | **3** |  |  | **253** | **3** |  |  |  |  |
|  |  |  |  |  |  |  |  |  |  | **299** | **1** |  |  |  |  |  |  | **378** | **1** |  |  |  |  | **330** | **2** |  |  | **255** | **2** |  |  |  |  |
|  |  |  |  |  |  |  |  |  |  | **302** | **1** |  |  |  |  |  |  | **390** | **1** |  |  |  |  |  |  |  |  | **257** | **5** |  |  |  |  |
|  |  |  |  |  |  |  |  |  |  | **305** | **3** |  |  |  |  |  |  | **393** | **2** |  |  |  |  |  |  |  |  | **260** | **3** |  |  |  |  |
|  |  |  |  |  |  |  |  |  |  | **315** | **1** |  |  |  |  |  |  | **412** | **1** |  |  |  |  |  |  |  |  | **262** | **7** |  |  |  |  |
|  |  |  |  |  |  |  |  |  |  | **317** | **2** |  |  |  |  |  |  | **425** | **1** |  |  |  |  |  |  |  |  | **264** | **5** |  |  |  |  |
|  |  |  |  |  |  |  |  |  |  | **336** | **2** |  |  |  |  |  |  | **428** | **2** |  |  |  |  |  |  |  |  | **269** | **10** |  |  |  |  |
|  |  |  |  |  |  |  |  |  |  | **349** | **1** |  |  |  |  |  |  | **438** | **2** |  |  |  |  |  |  |  |  | **271** | **21** |  |  |  |  |
|  |  |  |  |  |  |  |  |  |  | **352** | **1** |  |  |  |  |  |  | **442** | **1** |  |  |  |  |  |  |  |  | **273** | **12** |  |  |  |  |
|  |  |  |  |  |  |  |  |  |  | **358** | **3** |  |  |  |  |  |  |  |  |  |  |  |  |  |  |  |  | **275** | **5** |  |  |  |  |
|  |  |  |  |  |  |  |  |  |  | **382** | **1** |  |  |  |  |  |  |  |  |  |  |  |  |  |  |  |  | **277** | **3** |  |  |  |  |
|  |  |  |  |  |  |  |  |  |  | **387** | **1** |  |  |  |  |  |  |  |  |  |  |  |  |  |  |  |  | **279** | **1** |  |  |  |  |
|  |  |  |  |  |  |  |  |  |  | **393** | **1** |  |  |  |  |  |  |  |  |  |  |  |  |  |  |  |  | **282** | **4** |  |  |  |  |
|  |  |  |  |  |  |  |  |  |  | **396** | **1** |  |  |  |  |  |  |  |  |  |  |  |  |  |  |  |  | **284** | **2** |  |  |  |  |
|  |  |  |  |  |  |  |  |  |  | **439** | **2** |  |  |  |  |  |  |  |  |  |  |  |  |  |  |  |  | **286** | **2** |  |  |  |  |
|  |  |  |  |  |  |  |  |  |  | **443** | **1** |  |  |  |  |  |  |  |  |  |  |  |  |  |  |  |  | **291** | **1** |  |  |  |  |
|  |  |  |  |  |  |  |  |  |  | **452** | **1** |  |  |  |  |  |  |  |  |  |  |  |  |  |  |  |  | **293** | **1** |  |  |  |  |
|  |  |  |  |  |  |  |  |  |  |  |  |  |  |  |  |  |  |  |  |  |  |  |  |  |  |  |  | 295 | 1 |  |  |  |  |
|  |  |  |  |  |  |  |  |  |  |  |  |  |  |  |  |  |  |  |  |  |  |  |  |  |  |  |  | 297 | 1 |  |  |  |  |
|  |  |  |  |  |  |  |  |  |  |  |  |  |  |  |  |  |  |  |  |  |  |  |  |  |  |  |  | 301 | 2 |  |  |  |  |
|  |  |  |  |  |  |  |  |  |  |  |  |  |  |  |  |  |  |  |  |  |  |  |  |  |  |  |  | 303 | 1 |  |  |  |  |
|  |  |  |  |  |  |  |  |  |  |  |  |  |  |  |  |  |  |  |  |  |  |  |  |  |  |  |  | 305 | 1 |  |  |  |  |
|  |  |  |  |  |  |  |  |  |  |  |  |  |  |  |  |  |  |  |  |  |  |  |  |  |  |  |  | 317 | 1 |  |  |  |  |
|  |  |  |  |  |  |  |  |  |  |  |  |  |  |  |  |  |  |  |  |  |  |  |  |  |  |  |  | 329 | 1 |  |  |  |  |

^a^ SSR fragment size/alleles at analyzed loci

N, Number of isolates belong to the SSR fragment size or alleles.

**Supplementary File 2:** Direct sequencing of smallest and largest alleles from AF-16 and AF-42 showed that the large variation in fragment size is attributable to variation in repeat copy number


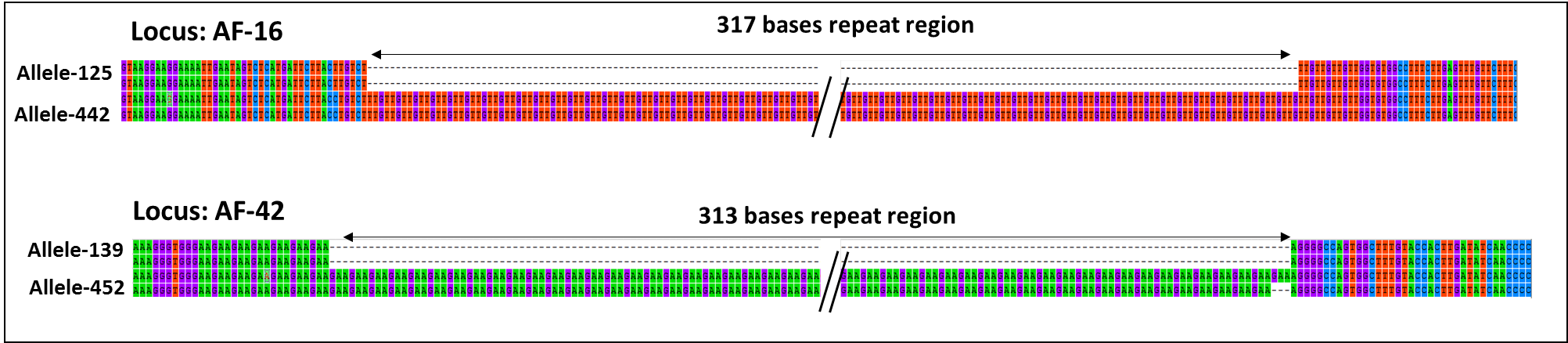


**Supplementary File 3:** Frequencies of clonal groups (closer related haplotype based on eBurst analysis) across 2408 isolates collected from ten agricultural soil cropped to maize in ten agricultural areas in southern, south-eastern, and south-western Kenya.


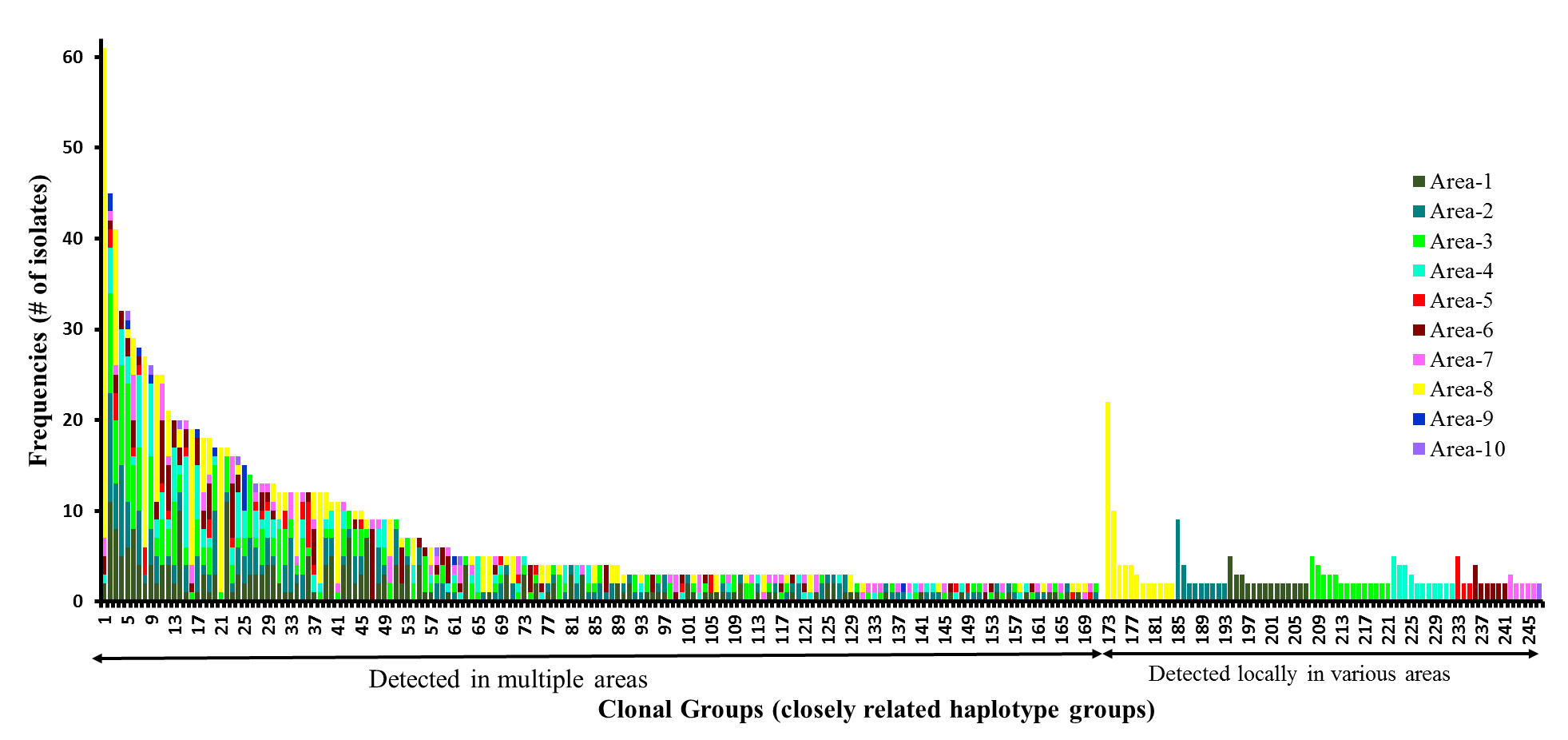


**Supplementary File 4:** Bayesian information criteria (BIC) supported three genetic clusters in Discriminant analyses of principal components (DAPC).This plot reflects the minimum number of clusters at 3 after which the BIC decreases by a negligible amount


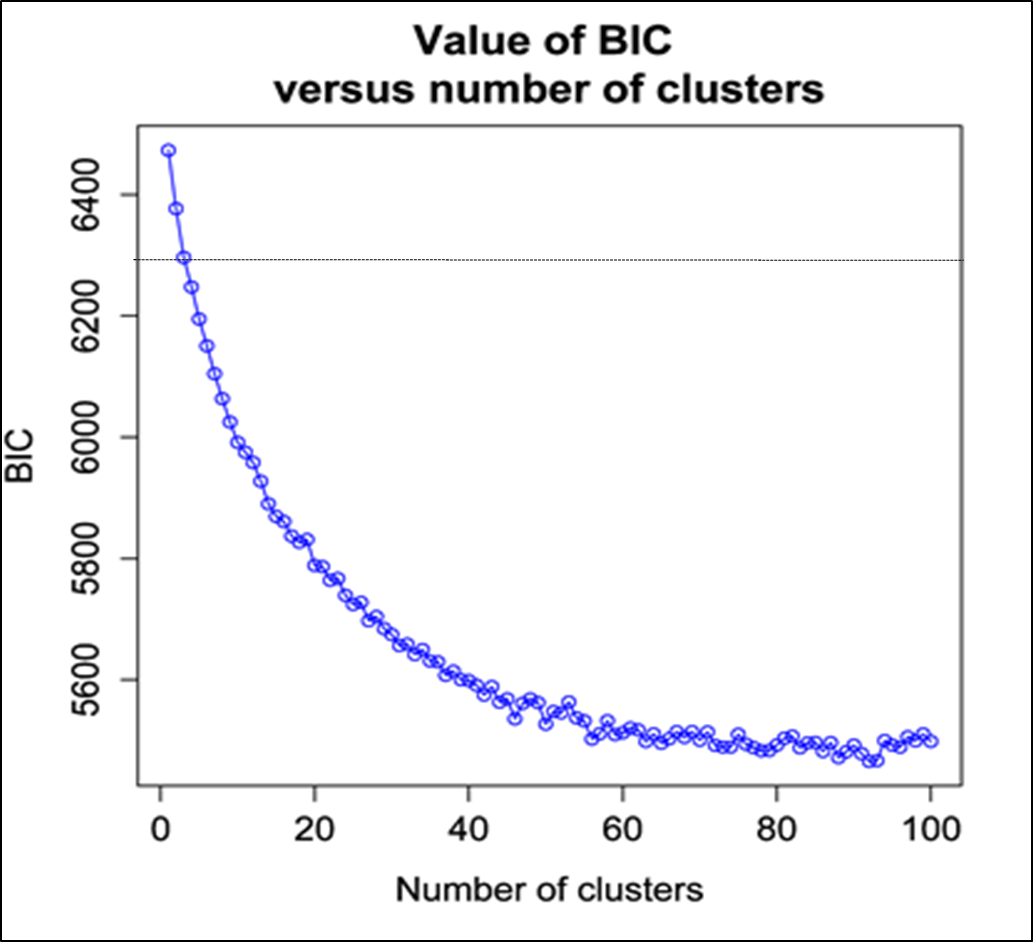


**Supplementary File 5:** Correlation between sample size (number of isolates) and percent isolates with a local distribution (belonging to private haplotypes)

**Supplementary File 6:** Standardized index of association, $\bar{r}$*_d_* as the measure of multilocus genotypic linkage disequilibrium (LD) across **a.** the members from 246 eBurst group (a single individual were chosen randomly from each of eBurst group); **b.** the members from 246 eBurst group + singletons. *P* < 0.01 indicates significant LD (at 99.9% level). The observed $\bar{r}$*_d_* for each of the DAPC lineages falls outside of the distribution expected under free recombination. N, number of isolates.


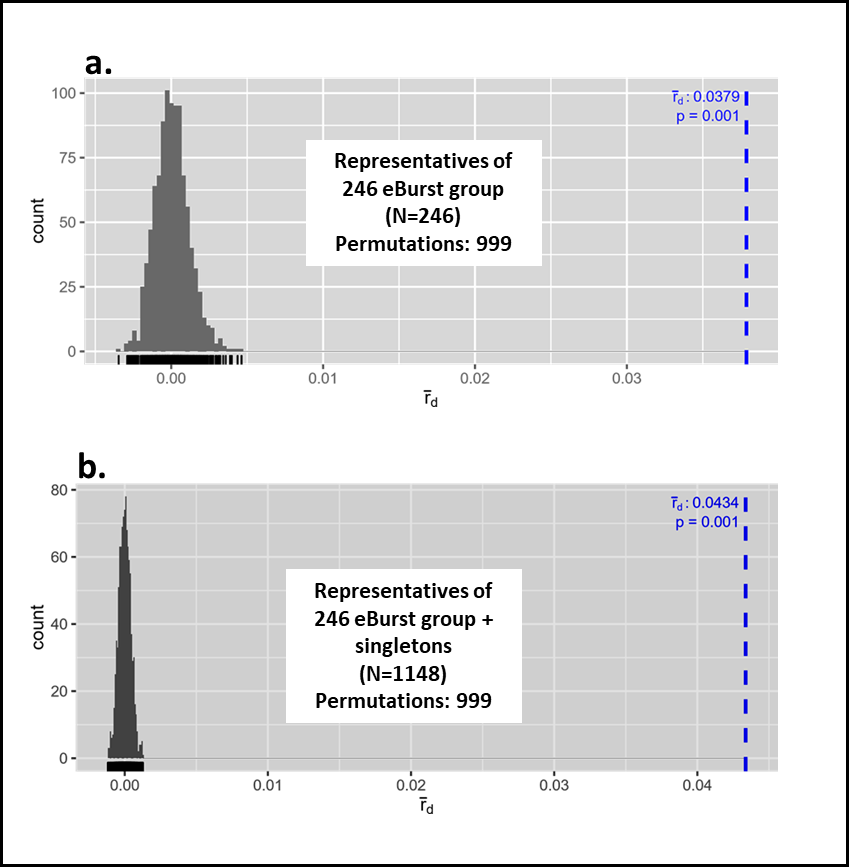


**Supplementary File 7:** Standardized index of association, $\bar{r}$*_d_* as the measure of multilocus genotypic linkage disequilibrium (LD) across **a.** the members from 31 eBurst group for DAPC-1 (a single individual were chosen randomly from each of eBurst group); **b.** the members from 31 eBurst group + singletons. *P* < 0.01 indicates significant LD (at 99.9% level). The observed $\bar{r}$*_d_* for each of the DAPC lineages falls outside of the distribution expected under free recombination. N, number of isolates.


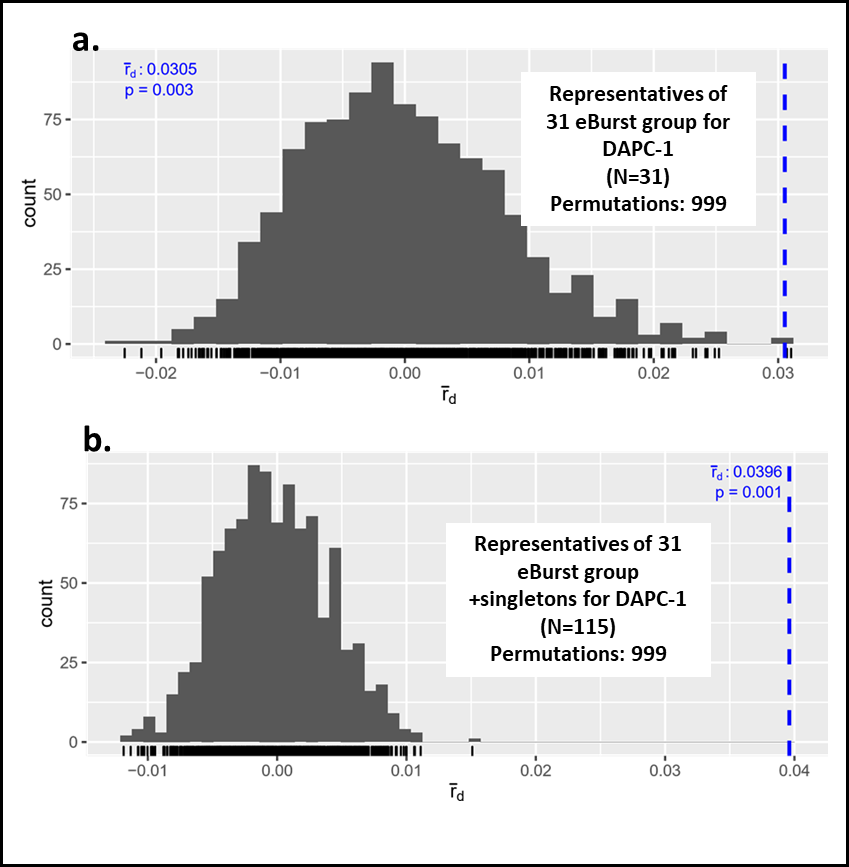


**Supplementary File 8:** Standardized index of association, $\bar{r}$*_d_* as the measure of multilocus genotypic linkage disequilibrium (LD) across **a.** the members from 160 eBurst group for DAPC-2 (a single individual were chosen randomly from each of eBurst group); **b.** the members from 160 eBurst group + singletons. *P* < 0.01 indicates significant LD (at 99.9% level). The observed $\bar{r}$*_d_* for each of the DAPC lineages falls outside of the distribution expected under free recombination. N, number of isolates.

**
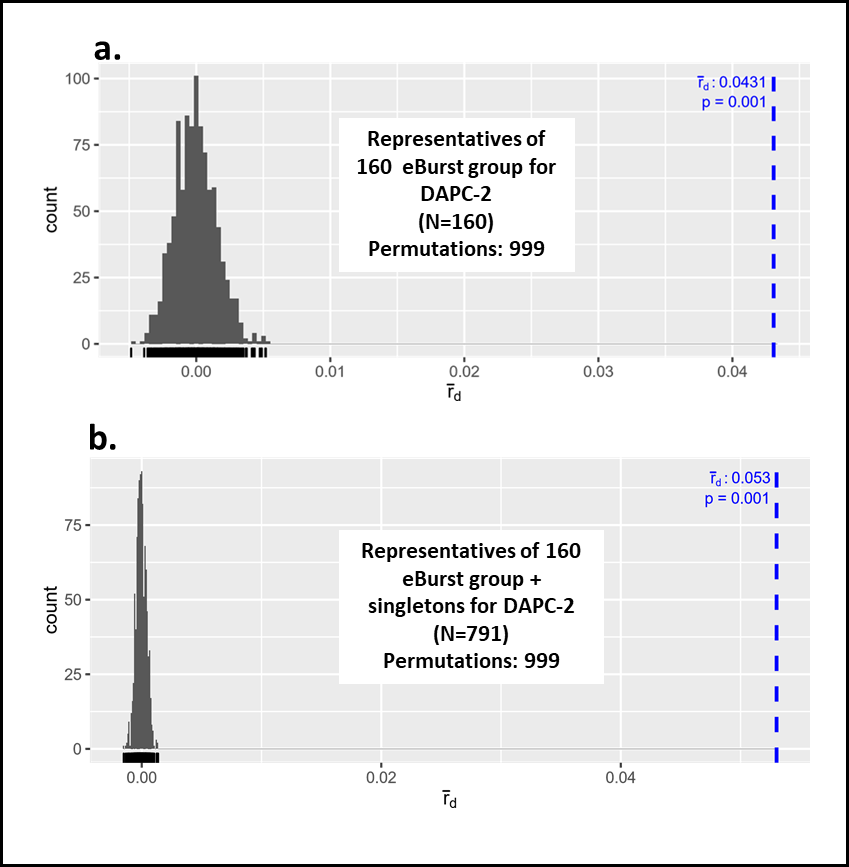
**

**Supplementary File 9:** Standardized index of association, $\bar{r}$*_d_* as the measure of multilocus genotypic linkage disequilibrium (LD) across **a.** the members from 55 eBurst group for DAPC-3 (a single individual were chosen randomly from each of eBurst group); **b.** the members from 55 eBurst group + singletons. *P* < 0.01 indicates significant LD (at 99.9% level). The observed $\bar{r}$*_d_* for each of the DAPC lineages falls outside of the distribution expected under free recombination. N, number of isolates.


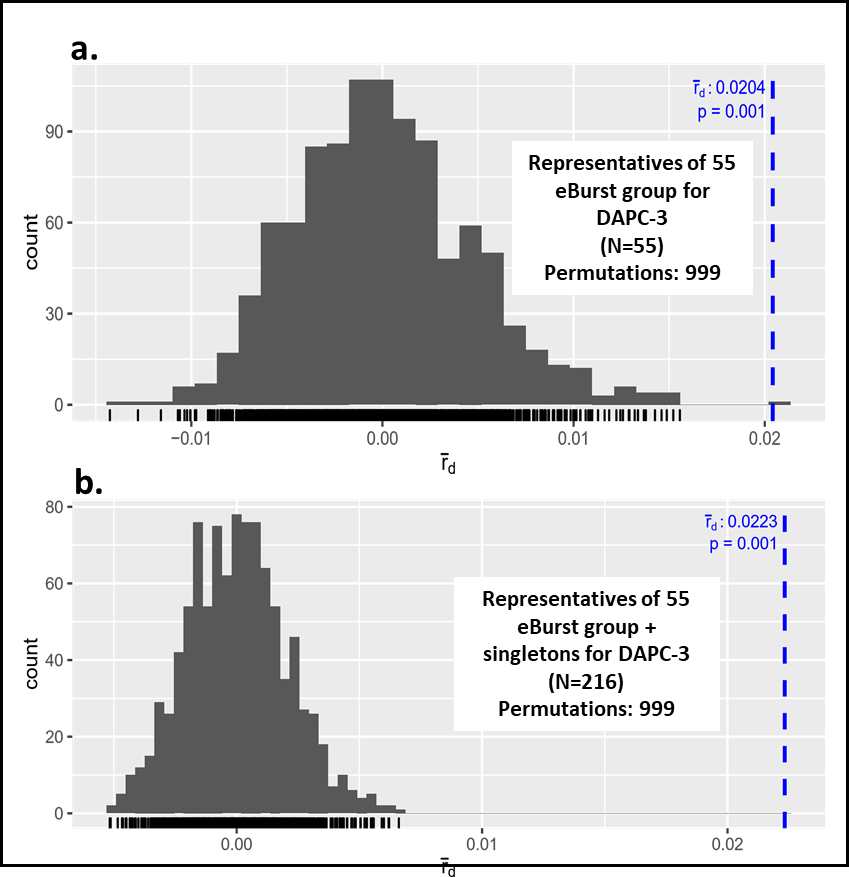

Supplement: Suplementary File-Islam [file mmc1.docx]
